# Mobilization of the nonconjugative virulence plasmid from hypervirulent *Klebsiella pneumoniae*

**DOI:** 10.1186/s13073-021-00936-5

**Published:** 2021-07-22

**Authors:** Yanping Xu, Jianfeng Zhang, Meng Wang, Meng Liu, Guitian Liu, Hongping Qu, Jialin Liu, Zixin Deng, Jingyong Sun, Hong-Yu Ou, Jieming Qu

**Affiliations:** 1 Department of Pulmonary and Critical Care Medicine, Ruijin Hospital, Shanghai Jiao Tong University School of Medicine, Shanghai 200025, China; Institute of Respiratory Diseases, Shanghai Jiao Tong University School of Medicine, Shanghai 200025, China; Shanghai Key Laboratory of Emergency Prevention, Diagnosis and Treatment of Respiratory Infectious Diseases, Shanghai, 200025 China; 2 State Key Laboratory of Microbial Metabolism, Joint International Laboratory on Metabolic & Developmental Sciences, School of Life Sciences & Biotechnology, Shanghai Jiao Tong University, Shanghai, 200030 China; 3 Department of Critical Care Medicine, Ruijin Hospital, Shanghai Jiao Tong University School of Medicine, Shanghai, 200025 China; 4 Department of Laboratory Medicine, Ruijin Hospital, Shanghai Jiao Tong University School of Medicine, Shanghai, 200025 China; 5 Department of Clinical Microbiology, Ruijin Hospital, Shanghai Jiao Tong University School of Medicine, Shanghai, 200025 China

**Keywords:** *Klebsiella pneumoniae*, Virulence plasmid, IncF plasmid, Conjugation, Mobilization

## Abstract

**Background:**

*Klebsiella pneumoniae*, as a global priority pathogen, is well known for its capability of acquiring mobile genetic elements that carry resistance and/or virulence genes. Its virulence plasmid, previously deemed nonconjugative and restricted within hypervirulent *K. pneumoniae* (hvKP), has disseminated into classic *K. pneumoniae* (cKP), particularly carbapenem-resistant *K. pneumoniae* (CRKP), which poses alarming challenges to public health. However, the mechanism underlying its transfer from hvKP to CRKP is unclear.

**Methods:**

A total of 28 sequence type (ST) 11 bloodstream infection-causing CRKP strains were collected from Ruijin Hospital in Shanghai, China, and used as recipients in conjugation assays. Transconjugants obtained from conjugation assays were confirmed by *Xba*I and S1 nuclease pulsed-field gel electrophoresis, PCR detection and/or whole-genome sequencing. The plasmid stability of the transconjugants was evaluated by serial culture. Genetically modified strains and constructed mimic virulence plasmids were employed to investigate the mechanisms underlying mobilization. The level of extracellular polysaccharides was measured by mucoviscosity assays and uronic acid quantification. An in silico analysis of 2608 plasmids derived from 814 completely sequenced *K. pneumoniae* strains available in GenBank was performed to investigate the distribution of putative helper plasmids and mobilizable virulence plasmids.

**Results:**

A nonconjugative virulence plasmid was mobilized by the conjugative plasmid belonging to incompatibility group F (IncF) from the hvKP strain into ST11 CRKP strains under low extracellular polysaccharide-producing conditions or by employing intermediate *E. coli* strains. The virulence plasmid was mobilized via four modes: transfer alone, cotransfer with the conjugative IncF plasmid, hybrid plasmid formation due to two rounds of single-strand exchanges at specific 28-bp fusion sites or homologous recombination. According to the in silico analysis, 31.8% (242) of the putative helper plasmids and 98.8% (84/85) of the virulence plasmids carry the 28-bp fusion site. All virulence plasmids carry the origin of the transfer site.

**Conclusions:**

The nonconjugative virulence plasmid in ST11 CRKP strains is putatively mobilized from hvKP or *E. coli* intermediates with the help of conjugative IncF plasmids. Our findings emphasize the importance of raising public awareness of the rapid dissemination of virulence plasmids and the consistent emergence of hypervirulent carbapenem-resistant *K. pneumoniae* (hv-CRKP) strains.

**Supplementary Information:**

The online version contains supplementary material available at 10.1186/s13073-021-00936-5.

## Background

*Klebsiella pneumoniae*, a common cause of hospital- and community-acquired infections, has increasingly gained public attention due to its capability of acquiring new plasmids and other mobile genetic elements that carry resistance- and/or virulence-associated genes [1–3]. *K. pneumoniae* strains can generally be classified into classical *K. pneumoniae* (cKP) and hypervirulent *K. pneumoniae* (hvKP) [4]. These two pathotypes can be distinguished by their disease profiles and genetic characteristics. cKP commonly causes infections in healthcare settings and carries plasmid(s) coding for antimicrobial resistance, and sequence type (ST) 11 carbapenem-resistant *K. pneumoniae* (CRKP) is the most prevalent *K. pneumoniae* strain in China. hvKP is typically associated with invasive diseases within the community and frequently harbours a virulence plasmid, which is the major pathogenic determinant of hypermucoviscosity and hypervirulence phenotypes [4], such as the well-documented virulence plasmid pLVPK of *K. pneumoniae* CG43 (GenBank accession number AY378100) [5] that encodes the mucoid regulators RmpA, aerobactin and salmochelin [6]. Potential biomarkers (including *peg-344*, *iroB*, *iucA*, *rmpA* and *rmpA2* as well as increased siderophore production in hvKP) were previously identified for the accurate differentiation of these two pathotypes [7]. Recently, *K. pneumoniae* isolates with genes conferring hypervirulence and multidrug resistance and even carbapenem resistance have increasingly emerged, which have caused bacteremia, metastatic infection and even death [8, 9]. These variants potentially developed in two directions [4, 9]: carbapenem-resistant hvKP (CR-hvKP), an hvKP acquiring a plasmid encoding carbapenemase, and hypervirulent carbapenem-resistant *K. pneumoniae* (hv-CRKP), a CRKP strain acquiring a virulence plasmid. Studies focusing on the transmission of self-transferable carbapenem-resistant plasmids have provided powerful support for the possibility that hvKP evolved into CR-hvKP [2, 10]. However, the virulence plasmids of *K. pneumoniae* are generally regarded as nonconjugative, and few studies have investigated the transfer of *K. pneumoniae* virulence plasmids.

Several recent studies have discovered resistance and virulence hybrid plasmids in *K. pneumoniae* of various sequence types, including hvKP clone types (e.g. ST23) [11] and cKP clone types (e.g. ST11, ST15, ST101 and ST147) [8, 12–14]. Some of these hybrid plasmids are combinations of conjugative resistance plasmids belonging to incompatibility group F (IncF) and virulence plasmids [2]. Yang* et al*. discovered such a hybrid plasmid in a clinical *Klebsiella variicola* strain and confirmed its self-transferable ability [15]. Another study observed homologous recombination between a virulence plasmid and an IncF1A plasmid in a clinical *K. pneumoniae* strain, which yielded a conjugative hybrid plasmid [16]. Li *et al*. reported that a non-pLVPK-like virulence plasmid could be transferred alone [17], but its helper plasmid and the corresponding mechanism remain to be uncovered. Notably, these studies focused on the existing conjugative hybrid plasmids in cKP or *K. variicola* but did not reveal the mechanism underlying the transmission of pLVPK-like nonconjugative virulence plasmids from hvKP to cKP, particularly ST11 CRKP [2].

We previously reported the hypervirulent *K. pneumoniae* strain RJF293 of capsular serotype K2 (accession number PRJNA307277) [18], which caused clinical metastatic infection, and confirmed its hypervirulence phenotype using a mouse lethality assay [19]. This strain carries the 224,263-bp virulence plasmid pRJF293 with high nucleotide sequence similarity (95% query coverage and 99% identities) to the classic virulence plasmid pLVPK. Here, we employed this pLVPK-like nonconjugative virulence plasmid and its variants to investigate the mechanism underlying virulence plasmid transfer. We found that the virulence plasmid could be transferred from hvKP to ST11 CRKP and *E. coli* strains with the help of a self-transferable IncF plasmid. We also identified four modes of virulence plasmid mobilization, including transfer with or without the conjugative IncF plasmid and fusion with the IncF plasmid via homologous recombination or two rounds of single-strand exchanges at specific 28-bp fusion sites. In this study, we also confirmed that virulence plasmid transfer from hvKP to CRKP could be limited by the overproduction of extracellular polysaccharides, which can be achieved by reducing extracellular polysaccharide production or employing *E. coli* strains as the intermediate vector. The in silico analysis revealed the wide distribution of putative conjugative helper plasmids and mobilizable virulence plasmids, which indicates that virulence plasmids might rapidly disseminate along with IncF plasmids that frequently carry carbapenemase genes.

## Methods

### Bacterial strains

For the conjugation assays (see below), the previously reported hvKP RJF293 [18] and its derivatives were employed as donor strains. Twenty-eight bloodstream infection-causing ST11 CRKP strains as well as ST11 CRKP HS11286 [20], its derivative HS11286-pKPHS2Δ*oriT*, and *E. coli* J53 and C600 were employed as recipient strains. All 28 ST11 CRKP strains were isolated from blood culture samples collected for routine clinical examinations of hospitalized patients admitted to Ruijin Hospital in Shanghai, China, from 2018 to 2019. The strains and plasmids used in this study are listed in Additional file 1: Table S1.

### Construction of genetically modified strains

For gene mutation or insertion, as described previously [10, 20], we replaced the target gene with the hygromycin B phosphotransferase gene (*hph*) flanked by flippase recognition sites (FRT) or inserted the *hph* gene via lambda red recombination. The *hph* gene conferring hygromycin resistance can be subsequently removed by Flp-FRT recombination. Details are provided in Additional file 2: Supplementary Methods. The primers used in this study are listed in Additional file 3: Table S2.

### Conjugation assay

Following overnight culture, both the donor and recipient strains were cultured at 220 rpm and 37°C to the logarithmic phase of growth (OD_600_ approximately 0.6) in lysogeny broth (LB) media. One millilitre of donor cells and recipient cells was washed with PBS, resuspended in 20 μl of 10 mM MgSO_4_, mixed and then inoculated on LB agar plates. After overnight culture at 37°C, the bacteria were resuspended and serially diluted in PBS and spread on antibiotic-containing LB agar plates for transconjugant selection. The antibiotic and the corresponding concentration used for each pair of conjugates are listed in Additional file 4: Table S3. The transconjugants were further validated by *Xba*I and S1 nuclease pulsed-field gel electrophoresis (PFGE) combined with PCR detection. According to the colony-forming unit (CFU) count on the serial dilution plates containing corresponding antibiotics, the conjugation frequency was calculated as the ratio of transconjugants to recipients.

### Whole-genome sequencing (WGS) and annotation

The genomic DNA of RJBSI76, RJBSI76-pV, J53-p1-pV-hybrid-1 and XL10-pF-pV-hybrid-1 was extracted and then sequenced using the combination of the 150-bp paired-end Illumina NovaSeq 6000 platform and the PacBio RSII single-molecule long-read sequencing platform. The trimmed and filtered reads were de novo assembled using Canu 2.0 [21]. The genome sequences are deposited in the National Center for Biotechnology Information (NCBI) BioProject repository under the accession numbers PRJNA681750 [22], PRJNA682095 [23], PRJNA692573 [24] and PRJNA692574 [25]. The genomic data were annotated with Prokka 1.1.3 [26]. PlasmidFinder 2.1 [27] was used to determine the plasmid incompatibility types, and Kleborate [28] was used to determine the sequence type of the strains. The putative virulence factors, antibiotic resistance determinants, insertion sequences (ISs) and other mobile genetic elements were predicted using VRprofile [29]. The conjugative transfer-related modules of the plasmids, including the relaxase gene, the type IV coupling protein (T4CP) gene and the *tra* gene cluster for the type IV secretion system (T4SS), were detected by oriTfinder [30]. Alignments of the plasmid sequences were performed using BLAST Ring Image Generator (BRIG) [31] and Easyfig [32].

### Construction of the mimic virulence plasmid

First, the origin of transfer (*oriT*) region of the virulence plasmid pRJF293HA was detected using the BLASTn searches against the back-end database of oriTfinder, oriTDB [30], with a high BLAST *E*-value of 1.0. The subsequent manual curation of the conserved nick site (*nic*) and the flanking putative inverted repeats (IRs) located within the *oriT* region was performed using MEME-MAST [33] and Vmatch (http://vmatch.de/), respectively. The predicted *oriT* of pRJF293HA was amplified, digested with *BamH*I and *Hind*III and then inserted into the plasmid pACYC184-Apr. The constructed plasmid pACYC184-Apr-*oriT*_RJF293HA_ was introduced into the *E. coli* strains XL10-pF and C600-p1 as well as their genetically modified strains with calcium chloride treatment. The transfer of the mimic virulence plasmid pACYC184-Apr-*oriT*_RJF293HA_ was validated by Sanger sequencing of the PCR product and PFGE using *Xba*I restriction.

### Plasmid stability in transconjugants

A single colony of each transconjugant was picked up from the freshly streaked agar plate and inoculated into fresh LB broth. Serial culture of each purified transconjugant was performed for 2 weeks. Ten microlitres of the bacterial suspension was transferred to 10 ml of fresh LB broth every 12 h. The plasmid stability was assessed by streaking each subculture of transconjugants on fresh LB agar plates and randomly selecting three single colonies for antibiotic resistance verification and PCR detection of the *iroB* gene of the virulence plasmid and the *traE* gene of the IncF plasmid. S1 nuclease-PFGE was performed with one of the three validated colonies.

### Mucoviscosity assay

As previously described, the mucoviscosity was determined using a sedimentation assay with some modifications [34]. Overnight cultures of *K. pneumoniae* strains grown in LB broth were subcultured to an OD_600_ of 0.2 in fresh LB broth and grown at 37°C for 4 h. The bacterial cultures were normalized to an OD_600_ of 1.0/ml and aspirated to 2 ml for sedimentation at 1000 ×*g* or 2500 × *g* for 5 min. The top 200 μl of the supernatant was carefully removed without disturbing the pellet for OD_600_ measurement.

### Capsule extraction and quantification assay

The extraction and quantification of uronic acid were performed as previously described with some modifications [34]. Overnight cultures grown in LB broth were subcultured to an OD_600_ of 0.2 in fresh LB medium and grown at 37°C for 4 h. Five hundred microlitres of bacterial culture was mixed with 100 μl of 1% Zwittergent-100 mM citric acid, and the mixture was incubated for 20 min at 50°C. The cells were pelleted by centrifugation, and 300 μl of the supernatant was aspirated into 1.2 ml of absolute ethanol, incubated for 20 min at 4°C and centrifuged at maximum speed for 5 min. The pellet was dried, resuspended in 200 μl of distilled water and mixed with 1.2 ml of 12.5 mM sodium tetraborate in sulfuric acid, and the mixture was incubated for 5 min at 100°C and then cooled on ice for 10 min. The absorbance at 520 nm was measured after the addition of 20 μl of 0.15% 3-phenylphenol in 0.5% NaOH. The glucuronic acid content was determined according to the standard curve for glucuronic acid and expressed as micrograms per OD unit.

### Bioinformatic analysis of sequenced plasmids of *K. pneumoniae*

As of January 28, 2021, 814 completely sequenced *K. pneumoniae* strains, including 2608 plasmids, were available in GenBank, and the accession numbers and genetic characteristics of these 2608 *K. pneumoniae* plasmids are listed in Additional file 5: Table S4. Plasmids that contain the oriTfinder-predicted *oriT* region and genes encoding relaxase, T4CP and T4SS were defined as putative conjugative plasmids and were included in the subsequent analysis. The sequenced plasmids containing the full-length *rmpA* (or *rmpA2*) gene and the *iuc* gene cluster were defined as putative virulence plasmids because the presence of these genes was sufficient for the host strain to exhibit some extent of hypervirulence, as previously reported [4]. The details of the selected putative conjugative plasmids and putative virulence plasmids are provided in Additional file 6: Table S5 and Additional file 7: Table S6, respectively. The 28-bp fusion site was identified using the matchPattern function in the Biostrings R package with the specific sequence 'AGATCCGNAANNNNNNNNTTNCGGATCT'. The *oriT* region and genes encoding relaxase, T4CP and T4SS were predicted using oriTfinder [30]. The sequence type of the strains, antimicrobial resistance gene and virulence gene were determined using Kleborate [28]. PlasmidFinder 2.1 [27] was used to determine the plasmid replicon types. The phylogenetic analysis was constructed using OrthoMCL [35], and the tree was drawn using iTOL [36].

### Statistical analysis

The data are presented as the means ± standard deviations (SDs) based on three independent experiments. The difference in average values between the two groups was assessed by unpaired two-sided Student’s *t*-test. A *P* value less than 0.05 was considered to indicate significance. The data analyses were performed using the R package (https://www.r-project.org/).

## Results

### Transfer of the nonconjugative virulence plasmid from hvKP to CRKP and further to *E. coli*

**Fig. 1:**
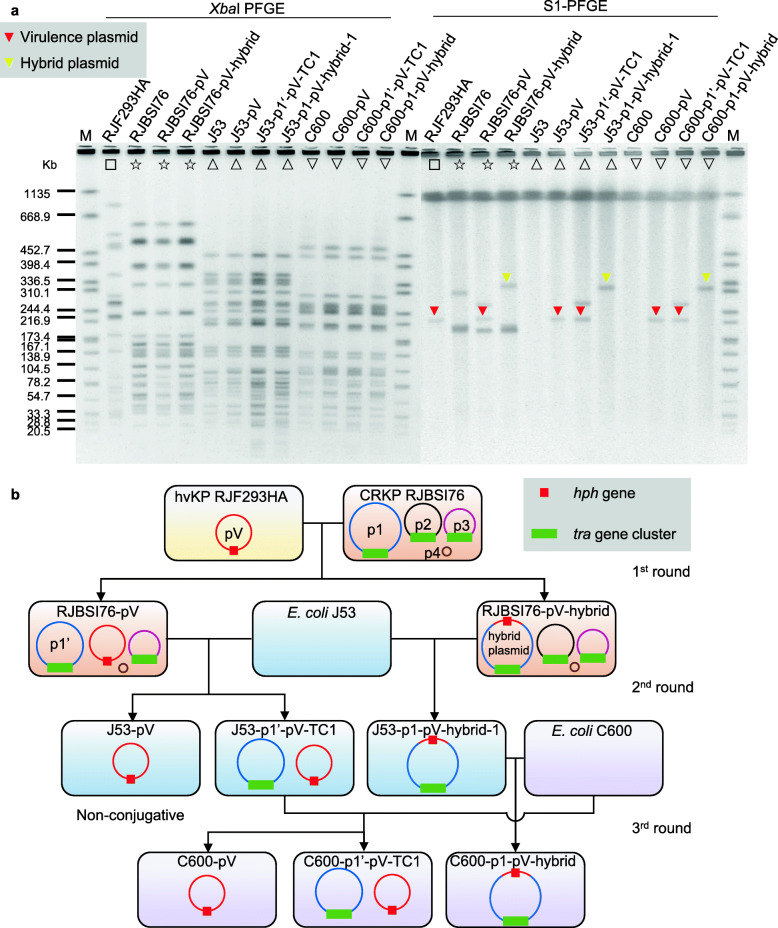
Transfer of the nonconjugative virulence plasmid from hvKP to carbapenem-resistant *K. pneumoniae* and *E. coli.* **a** *Xba*I PFGE and S1-PFGE of *K. pneumoniae* and *E. coli* transconjugants and their parental strains. Full details of the strains are provided in Additional file 1: Table S1. M represents the molecular weight marker *Salmonella* serotype Braenderup H9812 strain. Red triangles denote the virulence plasmid pRJF293HA. Yellow triangles denote the hybrid plasmids. Strains with the same symbol on the PFGE image represent progeny derived from the same parental strain. **b** Schematic diagram of the conjugation assays. The red square denotes the hygromycin B resistance gene *hph* tag on the virulence plasmid pRJF293HA. The green rectangle denotes the *tra* gene cluster encoding a T4SS on three IncF plasmids derived from RJBSI76, pRJBSI76-1 (p1), pRJBSI76-2 (p2) and pRJBSI76-3 (p3). The plasmid p1 in transconjugant RJBSI76-pV became smaller (p1’). Full details of the above-mentioned plasmids are shown in Additional file 2: Figs. S1-S5

To clarify the mechanism underlying virulence plasmid mobilization, RJF293C containing the pLVPK-like nonconjugative virulence plasmid pRJF293C was employed as the donor. Twenty-eight bloodstream infection-causing ST11 CRKP strains as well as ST11 CRKP HS11286 [20], its derivative HS11286-pKPHS2Δ*oriT*, and *E. coli* J53 and C600 were employed as the individual recipients for conjugation. However, the transmission of pRJF293C was not observed in each conjugation pair. Because the *rmpA* gene contributes to the overproduction of extracellular polysaccharides, which might inhibit plasmid transmission [37–39], the donor was switched to the reduced extracellular polysaccharide mutant *rmpA*-deficient RJF293HA (RJF293Δ*rmpA::hph*) for the following three rounds of conjugation (Fig. 1).

In the first round, the nonconjugative virulence plasmid pRJF293HA (also represented as pV in this study) was only transferred into CRKP RJBSI76. The obtained virulence plasmid pRJF293HA in the transconjugants RJBSI76-pV and RJBSI76-pV-hybrid showed two different patterns: (i) kept as a single plasmid and (ii) fused with pRJBSI76-1 (represented as p1) to yield a hybrid plasmid (named p1-pV-hybrid-1) (Fig. 1; Table 1). The virulence plasmids in both patterns were stable after serial cultures (Additional file 2: Fig. S1). The WGS of the transconjugant RJBSI76-pV suggested that the shorter plasmid pRJBSI76-pV-1 (represented as p1’) might be the result of two crossover homologous recombination events between pRJBSI76-1 and pRJF293HA (Additional file 2: Fig. S2).

**Table 1:** Conjugation frequency of the virulence plasmid pRJF293HA and its derivatives

Transconjugant	Donor	Recipient	Conjugation frequency
RJBSI76-pV^a^ (*iroB*^+^)	RJF293HA (pV-harbouring, *iroB*^+^)	RJBSI76 (p1-harbouring)	(1.01 ± 0.30) × 10^−7^
RJBSI76-pVC (*iroB*^+^, *rmpA*^+^)	C600-p1’-pVC (*iroB*^+^, *rmpA*^+^)	RJBSI76	(5.59 ± 2.79) × 10^−6^
J53-pV^b^ (*iroB*^+^)	RJBSI76-pV (*iroB*^+^)	J53	(1.43 ± 0.74) × 10^−4^
J53-pVC^c^ (*iroB*^+^, *rmpA*^+^)	RJBSI76-pVC (*iroB*^+^, *rmpA*^+^)	J53	(3.03 ± 1.46) × 10^−7^
J53-p1-pV-hybrid-1 (*iroB*^+^)	RJBSI76-pV-hybrid (*iroB*^+^)	J53	(4.58 ± 1.48) × 10^−5^
C600-p1-pV-hybrid (*iroB*^+^)	J53-p1-pV-hybrid-1 (*iroB*^+^)	C600	(2.02 ± 0.22) × 10^−1^
C600-pV^d^ (*iroB*^+^)	J53-p1’-pV-TC1 (*iroB*^+^)	C600	(4.18 ± 1.47) × 10^−2^
J53-p1	RJBSI76	J53	(2.38 ± 0.50) × 10^−3^
J53-p1’-pV-TC2^e^ (*iroB*^+^)	RJF293HA (pV-harbouring, *iroB*^+^)	J53-p1	(2.22 ± 0.45) × 10^−7^
C600-p1	J53-p1	C600	(1.88 ± 0.56) × 10^−2^
C600-p1’-pV-TC2 (*iroB*^+^)	RJF293HA (pV-harbouring, *iroB*^+^)	C600-p1	(5.53 ± 0.63) × 10^−6^
C600-p1’-pVC (*iroB*^+^, *rmpA*^+^)	RJF293C (pVC-harbouring, *iroB*^+^, *rmpA*^+^)	C600-p1	(5.83 ± 3.49) × 10^−8^
J53-pF-pV-TC1 (*iroB*^+^)	XL10-pF	J53-pV (*iroB*^+^)	(4.19 ± 0.21) × 10^−2^
XL10-pF-pV-TC1^f^ (*iroB*^+^)	J53-pV (*iroB*^+^)	XL10-pF	(1.04 ± 0.57) × 10^−7^
XL10-pV^g^ (*iroB*^+^)	J53-pF-pV-TC1 (*iroB*^+^)	XL10	(2.65 ± 1.33) × 10^−6^
XL10-pF-TC	J53-pF-pV-TC1 (*iroB*^+^)	XL10	(2.71 ± 1.04) × 10^−3^
J53-pF-pV-TC2^h^(*iroB*^+^)	XL10-pF-pV-hybrid-1 (*iroB*^+^)	J53	(6.74 ± 0.72) × 10^−2^
J53-pV_WT_^i^ (*iroB*^+^, *rmpA*^+^)	RJF293-pF (pV_WT_-harbouring, *iroB*^+^, *rmpA*^+^)	J53	(2.04 ± 0.36) × 10^−6^
HS11286-pF-pV_WT_-1^j^ (*iroB*^+^, *rmpA*^+^)	J53-pF-pV_WT_-hybrid	HS11286-pKPHS2Δ*oriT*	(1.46 ± 0.21) × 10^−5^
HS11286-pF-pV_WT_-2^k^ (*iroB*^+^, *rmpA*^+^)	J53-pF-pV_WT_	HS11286-pKPHS2Δ*oriT*	(1.68 ± 0.28) × 10^−9^
J53-pFΔ*oriT*-p1^l^	XL10-pFΔ*oriT*-p1	J53	(4.04 ± 0.52) × 10^−7^

^a^Included RJBSI76-pV and RJBSI76-pV-hybrid. ^b^Included J53-pV and J53-p1’-pV-TC1. ^c^Included J53-pVC and J53-p1’-pVC. ^d^Included C600-pV and C600-p1’-pV-TC1. ^e^Included J53-p1’-pV-TC2 and J53-p1-pV-hybrid-2. ^f^Included XL10-pF-pV-TC1 and XL10-pF-pV-hybrid-1. ^g^Included XL10-pV, XL10-pF-pV-TC2 and XL10-pF-pV-hybrid-2. ^h^Included J53-pF-pV-TC2 and J53-pF-pV-hybrid. ^i^Included J53-pV_WT_ and J53-pF-pV_WT_-hybrid and J53-pF-pV_WT_. ^j^Included HS11286-pF-pV_WT_-1 and HS11286-pF-pV_WT_-hybrid-1. ^k^Included HS11286-pF-pV_WT_-2 and HS11286-pF-pV_WT_-hybrid-2. ^l^Included J53-pFΔ*oriT*-p1 and J53-pFΔ*oriT*-p1-hybrid

In the second round, pRJF293HA could be further transferred from transconjugant RJBSI76-pV to *E. coli* J53 accompanied or not accompanied by the shorter plasmid p1’. The hybrid plasmid p1-pV-hybrid-1 in the transconjugant RJBSI76-pV-hybrid was transferred as a cointegrate. Similar findings were also observed in the third round (Fig. 1; Table 1). Notably, in the absence of p1’, the virulence plasmid in transconjugant J53-pV could not be transferred (Fig. 1). The WGS of the transconjugant J53-p1-pV-hybrid-1 suggested that the hybrid plasmid was a recombinant of pRJF293HA and pRJBSI76-1 with a 22-kb DNA fragment of pRJF293HA containing *hph* and *iroBCDN* inserted into the backbone of the IncF plasmid pRJBSI76-1 (Additional file 2: Fig. S3).

Overall, the nonconjugative virulence plasmid could be transferred from hvKP to ST11 CRKP and further to *E. coli* with or without the formation of the hybrid plasmid that emerged from homologous recombination.

### Mobilization of the pLVPK-like virulence plasmid with the help of the self-transferable IncF plasmid pRJBSI76-1

According to the WGS analysis, RJBSI76 contains three IncF plasmids and one small ColRNAI plasmid. The IncFIB plasmid pRJBSI76-1 (p1) carries known virulence genes (*rmpA2*, *iucABCDiutA*) and tellurite resistance genes (*terZABCDE* and *terW*) (Additional file 2: Fig. S4). The IncFII_K_/IncFIB_K_ plasmid pRJBSI76-2 (p2) encodes resistance to various classes of antibiotics (Additional file 2: Fig. S5a). The IncFII_pHN7A8_/IncR plasmid pRJBSI76-3 (p3) carries *bla*_KPC-2_ and other antimicrobial resistance genes (Additional file 2: Fig. S5b). Notably, all three IncF plasmids carry the *tra* gene clusters that encode the conjugative apparatus (Additional file 2: Fig. S4-S5).

**Fig. 2:**
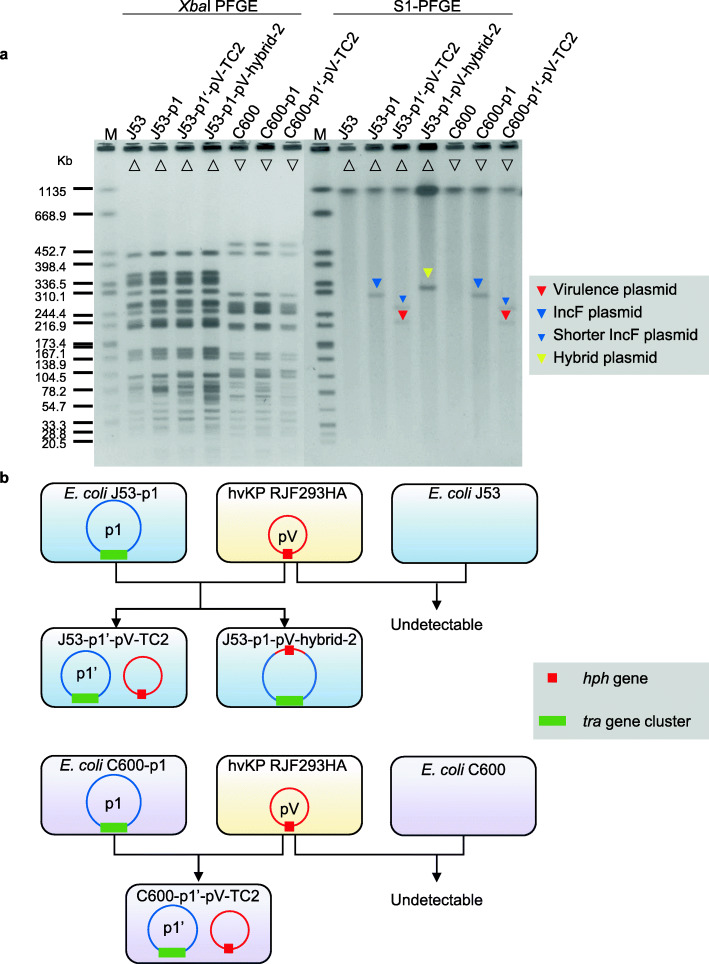
Mobilization of pRJF293HA with the help of the IncF plasmid pRJBSI76-1. **a** *Xba*I PFGE and S1-PFGE of *E. coli* transconjugants and their parental strains. Full details of the strains are provided in Additional file 1: Table S1. Red triangles denote the virulence plasmid pRJF293HA. Blue triangles denote the IncF plasmid p1 or its derivative. The yellow triangle denotes the hybrid plasmid. Strains with the same symbol on the PFGE image represent progeny derived from the same parental strain. **b**, **c** Schematic diagram of the conjugation assays. The red square denotes the *hph* tag on the virulence plasmid pRJF293HA. The green rectangle denotes the *tra* gene cluster on the IncFIB plasmid p1

To investigate which of the IncF plasmids of RJBSI76 plays a critical role in helping the transfer of pRJF293HA, we first evaluated the conjugative ability of three plasmids. Only pRJBSI76-1 was successfully transferred from RJBSI76 to J53, and the acquired pRJBSI76-1 helped the transmission of pRJF293HA from RJF293HA to J53-p1 with or without hybrid plasmid formation. Similarly, *E. coli* C600-p1 gained the virulence plasmid when conjugated with RJF293HA (Fig. 2; Table 1). These results showed that pRJBSI76-1 does play a critical role in the mobilization of virulence plasmid.

**Fig. 3:**
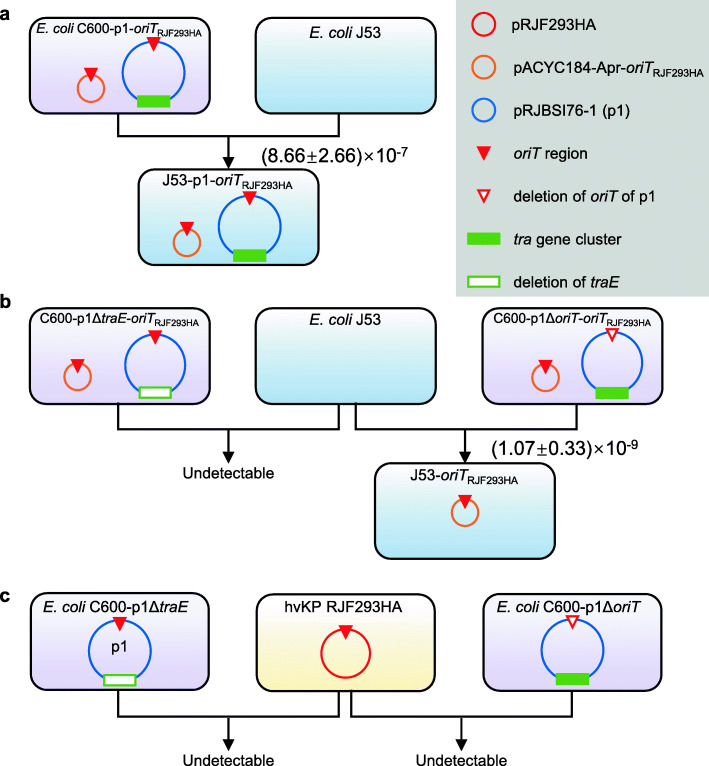
Schematic diagram of conjugation assays for the IncF plasmid pRJBSI76-1 and its nonconjugative derivatives. The red circle denotes the virulence plasmid pRJF293HA. The orange circle denotes the recombinant plasmid pACYC184-Apr-*oriT*_RJF293HA_. The blue circle denotes the IncF plasmid pRJBSI76-1 (p1). The red triangle denotes the *oriT* region of pRJF293HA (*oriT*_RJF293HA_) or that of p1. The green rectangle denotes the *tra* gene cluster on the IncF plasmid p1. The hollow red triangle on p1 represents the deletion of the *oriT* region. The hollow green rectangle on p1 indicates that the gene *traE* was deleted

We also explored whether the conjugative transfer regions of the IncF plasmid pRJBSI76-1, including the *tra* genes and the *oriT* region, are involved in virulence plasmid mobilization [40]. We constructed a mimic virulence plasmid carrying the predicted *oriT* region of pRJF293HA, which was denoted pACYC184-Apr-*oriT*_RJF293HA_. The nucleotide sequence of the *oriT* regions of pRJF293HA and pRJBSI76-1 showed 100% identities (Additional file 2: Fig. S6). The empty vector pACYC184-Apr was nonconjugative but pACYC184-Apr-*oriT*_RJF293HA_ was transferred from C600-p1-*oriT*_RJF293HA_ to J53 (Fig. 3a), which suggested that pRJBSI76-1 could mobilize the nonconjugative mimic virulence plasmid containing the *oriT* of pRJF293HA. We then knocked out the *traE* gene that encodes an essential component of T4SS responsible for seeding the site of pilus assembly [41] or the predicted *oriT* region on pRJBSI76-1 in *E. coli* C600-p1. *traE-*deficient pRJBSI76-1 failed to mobilize pACYC184-Apr-*oriT*_RJF293HA_ (Fig. 3b). Interestingly, pACYC184-Apr-*oriT*_RJF293HA_ could also transfer alone with the help of *oriT*-deficient pRJBSI76-1 at a very low frequency (Fig. 3b), which indicated the rolling circle replication of pACYC184-Apr-*oriT*_RJF293HA_, and the subsequent transfer could be finished with the help of the conjugative apparatus of pRJBSI76-1, which was consistent with the previously reported mobilization utilizing the T4SS on the helper plasmid [42]. In addition, neither modified pRJBSI76-1 nor pRJF293HA was transferred in the conjugation between RJF293HA and C600-p1Δ*traE* or C600-p1Δ*oriT* (Fig. 3c).

The above data showed that the nonconjugative virulence plasmid pRJF293HA could be mobilized by the self-transferable IncF plasmid pRJBSI76-1 encoding a functional T4SS.

### Mobilization of the pLVPK-like virulence plasmid with the help of the conjugative IncF plasmid pOX38-Gen

**Fig. 4:**
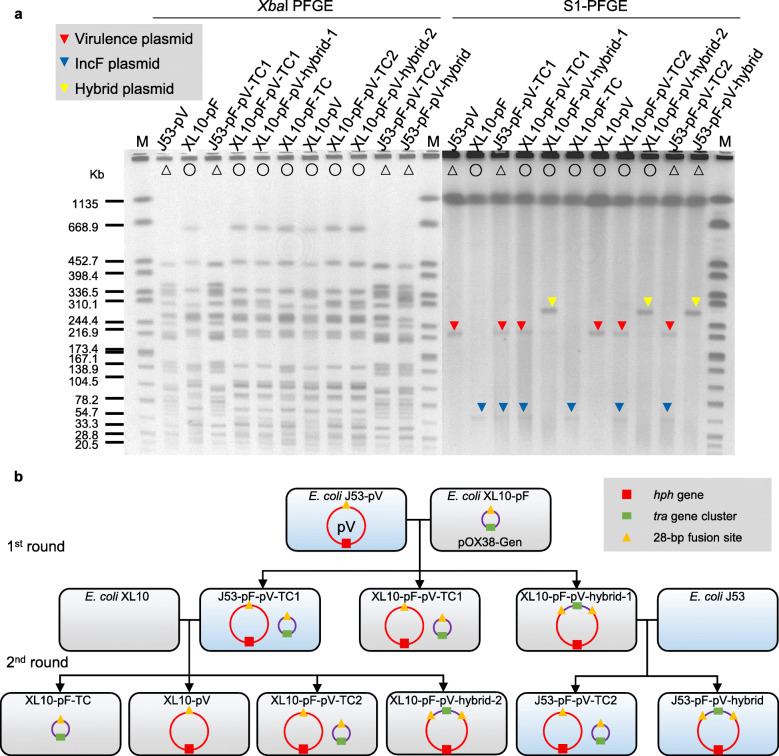
Mobilization of pRJF293HA with the help of the IncF plasmid pOX38-Gen. **a** *Xba*I PFGE and S1-PFGE of *E. coli* transconjugants and their parental strains. Full details of the strains are provided in Additional file 1: Table S1. Red triangles denote the virulence plasmid pRJF293HA. Blue triangles denote the IncF plasmid derivative pOX38-Gen. Yellow triangles denote the hybrid plasmid (full details are shown in Fig. 5). Strains with the same symbol on the PFGE image represent progeny derived from the same parental strain. **b** Schematic diagram of the conjugation assays. The red square denotes the *hph* tag on the virulence plasmid pRJF293HA. The green rectangle denotes the *tra* gene cluster coding for a T4SS on the IncF plasmid derivative pOX38-Gen. The orange triangles denote the specific 28-bp fusion site

Because the large IncF plasmid pRJBSI76-1 has a complicated structure, we employed the IncFIA plasmid derivative pOX38-Gen (also represented as pF) to further investigate the mechanism of pRJF293HA mobilization. The transmissions of both pOX38-Gen and pRJF293HA were observed in the conjugation between XL10-pF and J53-pV. Remarkably, two types of transconjugants contained virulence plasmids: pRJF293HA was fused with pOX38-Gen or remained unchanged (Fig. 4; Table 1).

**Fig. 5:**
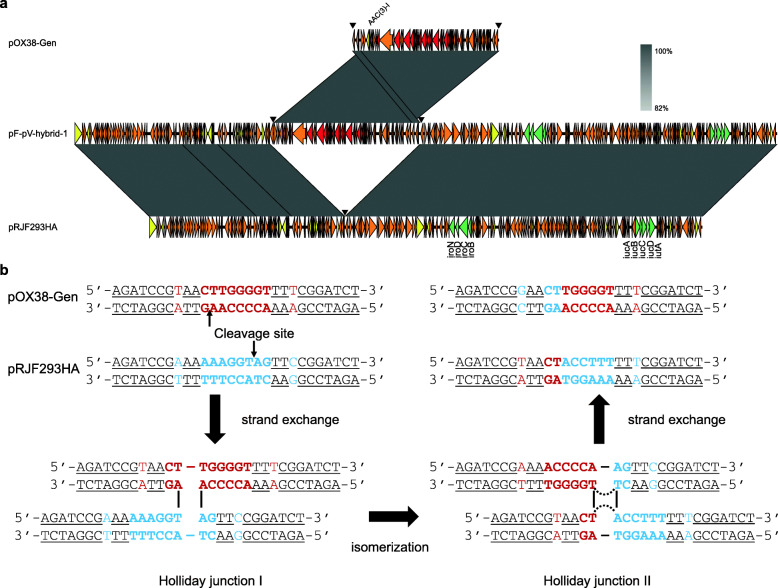
Hybrid plasmid formation via two rounds of single-strand exchanges. **a** Sequence alignments of pOX38-Gen, the hybrid plasmid in XL10-pF-pV-hybrid-1 and pRJF293HA. The figure was constructed using Easyfig. The black inverted triangle denotes the specific 28-bp fusion site where the whole sequence of pOX38-Gen was inserted into RJF293HA. Green, virulence genes. Red, *tra* genes. Yellow, IS elements. **b** Putative mechanism underlying the formation of the hybrid plasmid pF-pV-hybrid-1. This hybrid plasmid possibly emerged from two rounds of chain cleavages and exchanges between pOX38-Gen and pRJF293HA within the 28-bp homologous region, which consists of 10-bp inverted repeat sequences (underlined) and an 8-bp internal spacer region (bold). The arrowheads represent the supposed cleavage sites for recombination. The shared sequence is shown in black, the sequence specific to pOX38-Gen is shown in red and the sequence specific to pRJF293HA is shown in blue

In the conjugation between XL10 and J53-pF-pV-TC1 harbouring separate pOX38-Gen and pRJF293HA, various modes of pRJF293HA mobilization were detected: (i) fused with pOX38-Gen, (ii) transferred with pOX38-Gen and (iii) transferred alone. The hybrid plasmid of XL10-pF-pV-hybrid-1 could further be transferred to J53, which yielded transconjugants with or without hybrid plasmid resolution (Fig. 4; Table 1). The formation and resolution of the hybrid plasmid pF-pV-hybrid-1 were observed during serial passages (Additional file 2: Fig. S7), which suggested that the fusion event was reversible. A WGS analysis of XL10-pF-pV-hybrid-1 revealed that pRJF293HA was entirely integrated into pOX38-Gen between 28-bp fusion sites shared on both plasmids (Fig. 5). The results indicate that the specific regions consist of two conserved 10-bp inverted repeat sequences and a variable 8-bp internal spacer region where the recombinant junction was supposed to occur. It has been proposed that two rounds of strand cleavage and exchange lead to the generation and resolution of a Holliday junction intermediate [43]. To validate the specific fusion event, conjugation between XL10-pFΔ*oriT*-p1 and J53 was performed. The nonconjugative *oriT*-deficient pOX38-Gen was mobilized by pRJBSI76-1 with or without a fusion event at 28-bp fusion sites (Additional file 2: Fig. S8).

To further investigate the mechanism underlying mobilization without hybrid plasmid formation, the mimic virulence plasmid and knockout strains were employed. The mimic virulence plasmid pACYC184-Apr-*oriT*_RJF293HA_ rather than the empty vector pACYC184-Apr could be mobilized by pOX38-Gen (Additional file 2: Fig. S9a). Both *oriT*-deficient pOX38-Gen and *traE*-deficient pOX38-Gen lost their conjugative ability and the transmission of pACYC184-Apr-*oriT*_RJF293HA_ or the virulence plasmid pRJF293HA was not detected (Additional file 2: Fig. S9b, c).

Altogether, these results showed that the nonconjugative virulence plasmid pRJF293HA containing *oriT* and the 28-bp fusion site could transfer alone, cotransfer with the IncF plasmid or fuse with the IncF plasmid at 28-bp fusion sites with the help of the IncF plasmid pOX38-Gen harbouring a functional T4SS.

### *E. coli* promoted indirect virulence plasmid transfer from hvKP with overproduced extracellular polysaccharides into CRKP

The *rmpA*-positive virulence plasmid pRJF293C (represented as pVC) was not transferred between RJF293C and RJBSI76 but was successfully transferred from RJF293C to C600-p1 and further to RJBSI76, leaving the *K. pneumoniae* transconjugant RJBSI76-pVC positive for the string test (Table 1; Additional file 2: Fig. S10). Compared with RJF293HA and RJBSI76-pV, RJF293C and RJBSI76-pVC less easily transferred the virulence plasmid to the *E. coli* strain, and these difficulties might be explained by significant increases in mucoviscosity and extracellular polysaccharide production (Additional file 2: Fig. S11).

Similarly, the virulence plasmid pRJF293 (also represented as pV_WT_) was not transferred between RJF293-pF and HS11286 or its derivative HS11286-pKPHS2Δ*oriT*, whereas *E. coli* J53 could serve as an intermediate vector to promote virulence plasmid transfer. pRJF293 was transferred from RJF293-pF to *E. coli* J53 alone or together with pOX38-gen with or without hybrid plasmid formation. The virulence plasmid was transferred from J53-pF-pV_WT_ and J53-pF-pV_WT_-hybrid to HS11286-pKPHS2Δ*oriT* via various modes, and significantly higher conjugation frequency was obtained with hybrid plasmid formation in the donor strain [(1.46 ± 0.21) × 10^−5^ versus (1.68 ± 0.28) × 10^−9^, *P =* 0.00058] (Table 1; Additional file 2: Fig. S12). The acquisition of pRJF293 also made HS11286-pKPHS2Δ*oriT* transconjugants hypermucoviscous.

The above-described results showed that the virulence plasmid could be transferred from conjugative IncF plasmid-containing hvKP to *E. coli*. The *E. coli* transconjugants containing both virulence and IncF plasmids, with or without hybrid plasmid formation, could further transfer the virulence plasmid or the hybrid plasmid to CRKP via different modes.

### Distribution of putative helper plasmids and mobilizable virulence plasmids among *K. pneumoniae*

Based on the above-described conjugation results and the previously reported requirements for helper plasmids [40], we hypothesized that conjugative plasmids are potential helper plasmids in *K. pneumoniae* and that virulence plasmids containing the *oriT* region or the specific 28-bp fusion site are mobilizable. We searched 2608 plasmids in 814 completely sequenced *K. pneumoniae* strains available in GenBank to investigate the distribution of putative helper plasmids and mobilizable virulence plasmids.

A total of 29.1% (760/2608) of plasmids are considered conjugative and have the ability to mobilize virulence plasmids containing the *oriT* region (Additional file 2: Fig. S13), and 31.8% (242) of these plasmids were found to carry the 28-bp fusion site and might have the potential to mobilize virulence plasmids containing the 28-bp fusion site, resulting in the formation of hybrid plasmids. A total of 2.2% (17) of the putative helper plasmids carry two or three 28-bp fusion sites, whose significance remains to be investigated. The putative conjugative plasmids exhibit a wide distribution range over the *K. pneumoniae* strains of various sequence types, and ST11 is the dominant clone type, followed by ST258, ST147, ST231 and others. Notably, 67.6% (514) of the putative helper plasmids were IncF plasmids and showed a higher percentage of plasmids containing 28-bp fusion sites than non-IncF-type plasmids (47.8% vs 5.7%). Significantly, 75.8% (576) of the predicted conjugative plasmids contained one or more antimicrobial resistance genes, and 23.8% (181) harboured carbapenemase genes, which suggested that *K. pneumoniae* strains with multidrug resistance and hypervirulence could potentially be generated by cotransfer of the virulence plasmids with the IncF plasmids encoding antimicrobial resistance.

A total of 85 *K. pneumoniae* virulence plasmids were extracted (Additional file 2: Fig. S14). Six virulence plasmids harbour at least one antimicrobial resistance gene. Six plasmids contain T4SS gene clusters, and the virulence plasmids were distributed in both cKP clone types (e.g. ST11, ST15 and ST383) and hvKP clone types (e.g. ST23, ST65, ST66 and ST86). All the virulence plasmids harbour the *oriT* region, and 84 of them have at least a specific 28-bp fusion site, which indicates that all the selected virulence plasmids can be considered mobilizable.

## Discussion

**Fig. 6:**
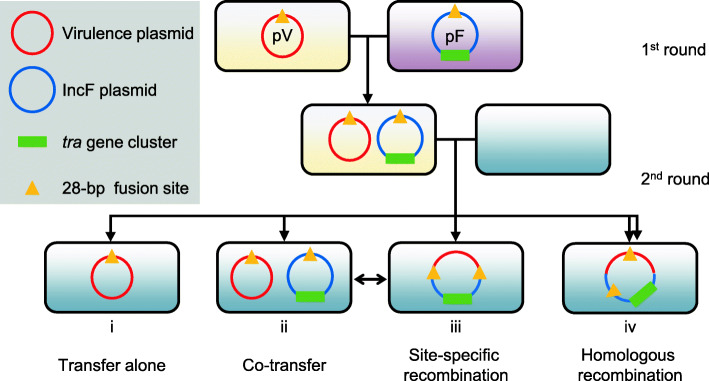
Proposed model of the mobilization of the pLVPK-like nonconjugative virulence plasmid of *K. pneumoniae*. The initial step of virulence plasmid transfer is the acquisition of a conjugative IncF plasmid, and this step is followed by virulence plasmid mobilization via four modes. The virulence plasmid could be transferred (i) alone, (ii) cotransferred with the IncF plasmid, (iii) fused with the IncF plasmid due to recombination at specific 28-bp fusion sites or (iv) fused with the IncF plasmid due to recombination in the homologous region. The green rectangle denotes the *tra* gene cluster coding for a T4SS on the IncF plasmid. The orange triangles denote the specific 28-bp fusion site

The pLVPK-like virulence plasmid of hvKP is generally considered to be nonconjugative and absent from cKP. Several studies conducted in recent years have reported emerging virulence plasmid-harbouring cKP strains [2, 8, 9], which has raised public concerns regarding the availability of an effective treatment for this type of threatening pathogen. However, the process of virulence plasmid transfer from hvKP to CRKP and the important elements essential for virulence plasmid transfer remain unclarified. Here, we demonstrated that under reduced extracellular polysaccharide-producing conditions or by employing *E. coli* intermediate strains, the pLVPK-like nonconjugative virulence plasmid could be transferred from hvKP to ST11 CRKP strains via four different modes (Fig. 6). The IncF plasmid, as a driving force in virulence plasmid mobilization, is reportedly prevalent in *Enterobacteriaceae* worldwide and carries a great variety of antimicrobial resistance genes, accounting for almost 40% of plasmid-borne carbapenemases [44, 45]. Attention should be given to the potential risks that antibiotic selection might also promote the dissemination of virulence plasmids and enrich hypervirulent multidrug-resistant cKP (hv-MDR-cKP) or hv-CRKP strains.

The virulence plasmid pRJF293HA in the presence of *oriT* was found to be transferred alone or cotransferred with the conjugative IncF plasmid. The reported virulence plasmids in ST11 or ST15 cKP strains might be obtained through the former two approaches [9, 17, 46]. The *oriT* sequence of the previously submitted virulence plasmid p17-16-vir was the same as that of pRJF293, and p17-16-vir was found to be transferred via mode i and mode iv [17] (Fig. 6). A functional T4SS is an indispensable element for helper plasmids, as has been verified by the loss of self-transferability and failure in the mobilization of virulence plasmids or mimic virulence plasmids after the deletion of *traE* on IncF plasmids. We also noticed that the helper plasmid could employ the *oriT* of the mobilized plasmid to complete the mobilization, which was consistent with a previous study [42].

The previously reported hybrid plasmids include those that emerged from homologous recombination in *K. pneumoniae* strains [16] and IS*26*-mediated incorporation in a *Salmonella enteritidis* strain [47]. Here, we identified a novel fusion event between a nonconjugative virulence plasmid and a self-transferable IncF plasmid due to site-specific recombination: mode iii. The specific 28-bp fusion site of pOX38-Gen was previously reported and named the replicon fusion site of F (*rfsF*) [43], within which recombination and resolution events were observed only between F plasmids via two rounds of single-strand exchanges [48]. Similarly, integration and resolution events occurred between the virulence plasmid pRJF293HA and the IncF plasmid pOX38-Gen at the specific 28-bp fusion sites. This fusion event was repeated between pRJBSI76-1 and pOX38-GenΔ*oriT* (Additional file 2: Fig. S8)*.* A more efficient virulence plasmid transfer was observed after hybrid plasmid formation at 28-bp fusion sites in the donor strain (Table 1; Additional file 2: Fig. S12), which might allow the mobilization of virulence plasmids by a broader set of IncF plasmids. Whether the various modes proposed in our study are universal in clinical settings and whether other mobilization modes exist require further large-scale surveillance studies on virulence plasmid transmission and evolution.

According to the in silico analysis of the completely sequenced *K. pneumoniae* available in GenBank, 29.1% of plasmids possess a conjugative apparatus and have the potential to serve as helper plasmids. We also found that all the virulence plasmids were mobilizable because they contained an *oriT* region with or without 28-bp fusion site(s). The 28-bp fusion sites were also distributed in 31.8% of the helper plasmids. These results alarmingly indicate that antibiotic abuse might contribute to the explosive dissemination of helper plasmids coding for antimicrobial resistance [49, 50] and promote the transfer of virulence plasmids and the emergence of hv-MDR-cKP or hv-CRKP.

The virulence plasmid was not transferred in the direct interactions between hypermucoviscous hvKP RJF293C and ST11 CRKP strains but was mobilized from *rmpA*-deficient mutant RJF293HA to CRKP RJBSI76. In addition, the interspecies transmission of pRJF293C was less frequent than that of pRJF293HA. Our findings are consistent with previous studies that reported that overproduced extracellular polysaccharides might serve as a barrier for plasmid transfer because they could conceal crucial attachment sites for the conjugative apparatuses, such as OmpA porin and lipopolysaccharide [37, 51, 52]. *E. coli* strains harbouring conjugative IncF plasmids could serve as the intermediate vector to deliver the virulence plasmid indirectly from hypermucoviscous hvKP into CRKP, resulting in the transconjugants hypermucoviscosity phenotype, which is usually associated with hypervirulence [4, 53]. The results imply that the microbial environment might influence virulence plasmid transmission by regulating extracellular polysaccharide production, and *E. coli* strains might also serve as a reservoir of the virulence plasmid of *K. pneumoniae*.

## Conclusions

Our study first confirmed the mobilization of a pLVPK-like nonconjugative virulence plasmid from hvKP into CRKP and *E. coli* strains with the help of a self-transferable IncF plasmid. The various mobilization modes observed in the study deepened our understanding of the transfer of virulence plasmids. Inappropriate antibiotic usage might boost the transfer of conjugative plasmids encoding antimicrobial resistance and the dissemination of nonconjugative virulence plasmids, which contributes to the ongoing emergence of *K. pneumoniae* with both antimicrobial resistance and hypervirulence.

## Supplementary Information

**Additional file 1.** Table S1. Strains and plasmids used in this study.

**Additional file 2. **Supplementary Methods. Figure S1. Plasmid stability of serial cultures of the *K. pneumoniae* transconjugants RJBSI76-pV and RJBSI76-pV-hybrid. Figure S2. Homologous recombination between the IncF plasmid pRJBSI76-1 and the virulence plasmid pRJF293HA. Figure S3. Sequence alignments of the IncF plasmid pRJBSI76-1, the hybrid plasmid p1-pV-hybrid-1 and the virulence plasmid pRJF293HA. Figure S4. Genetic structure of the IncFIB plasmid pRJBSI76-1 of the clinical CRKP strain RJBSI76. Figure S5. Genetic structure of the plasmids pRJBSI76-2 and pRJBSI76-3 of the clinical CRKP strain RJBSI76. Figure S6. Sequence alignment of three predicted *oriT* regions. Figure S7. Plasmid stability of serial cultures of the *E. coli* transconjugants J53-pF-pV-hybrid and J53-pF-pV-TC2. Figure S8. Validation of the fusion event at 28-bp fusion sites. Figure S9. Schematic diagram of the mobilization of a virulence plasmid by the conjugative IncF plasmid pOX38-Gen. Figure S10. Indirect transfer of the virulence plasmid from hvKP RJF293C to CRKP RJBSI76. Figure S11. The less-frequent transfer of pRJF293C was due to increased production of extracellular polysaccharides by the donor. Figure S12. Indirect transfer of the virulence plasmid from hvKP RJF293-pF to CRKP HS11286-pKPHS2Δ*oriT*. Figure S13. *In silico* analysis of 760 conjugative plasmids of the completely sequenced *K. pneumoniae* in GenBank. Figure S14. Mobilization potential of 85 virulence plasmids of the completely sequenced *K. pneumoniae* available in GenBank.

**Additional file 3.** Table S2. Oligonucleotides used in this study.

**Additional file 4.** Table S3. Antibiotics and corresponding concentrations used for each conjugation pair.

**Additional file 5. **Table S4. NCBI accession numbers and genetic characteristics of 2608 *K. pneumoniae* plasmids.

**Additional file 6. **Table S5. NCBI accession numbers and genetic characteristics of 760 putative conjugative plasmids of *K. pneumoniae*.

**Additional file 7. **Table S6. NCBI accession numbers and genetic characteristics of 85 putative mobilizable virulence plasmids of *K. pneumoniae*.

## Data Availability

The complete genome sequences of *K. pneumoniae* RJBSI76 and RJBSI76-pV, as well as *E. coli* J53-p1-pV-hybrid-1, were deposited in the NCBI BioProject repository under the accession numbers PRJNA681750 (https://www.ncbi.nlm.nih.gov/nuccore/?term=PRJNA681750) [22], PRJNA682095 (https://www.ncbi.nlm.nih.gov/nuccore/?term=PRJNA682095) [23], and PRJNA692573 (https://www.ncbi.nlm.nih.gov/nuccore/?term=PRJNA692573) [24]. The genome sequence of the hybrid plasmid pF-pV-hybrid-1 derived from *E. coli* strain XL10-pF-pV-hybrid-1 was deposited in the NCBI BioProject repository under the accession number PRJNA692574 (https://www.ncbi.nlm.nih.gov/nuccore/1968731931) [25]. The accession numbers of all the other sequences analysed during the current study are included in this manuscript and available in the NCBI Nucleotide database [54].

## Abbreviations

CFU: Colony-forming unit
cKP: Classic *Klebsiella pneumoniae*
CR-hvKP: Carbapenem-resistant hypervirulent *Klebsiella pneumoniae*
CRKP: Carbapenem-resistant *Klebsiella pneumoniae*
FRT: Flippase recognition sites
hv-CRKP: Hypervirulent carbapenem-resistant *Klebsiella pneumoniae*
hv-MDR-cKP: Hypervirulent multidrug-resistant classic *Klebsiella pneumoniae*
hvKP: Hypervirulent *Klebsiella pneumoniae*
IncF: Incompatibility group F
IR: Inverted repeat
IS: Insertion sequence
LB: Lysogeny broth
NCBI: National Center for Biotechnology Information
*nic*: Nick site at the origin of transfer (*oriT*)
*oriT*: Origin of transfer
PFGE: Pulsed-field gel electrophoresis
*rfsF*: Replicon fusion site of F
SD: Standard deviation
ST: Sequence type based on the multilocus sequence typing
T4CP: Type IV coupling protein
T4SS: Type IV secretion system
WGS: Whole-genome sequencing
