# Supplementary material for: Mobilization of the nonconjugative virulence plasmid from hypervirulent Klebsiella pneumoniae
Source: Genome Med. 2021 Jul 22;13:119. doi: 10.1186/s13073-021-00936-5 (PMC8299605; doi:10.1186/s13073-021-00936-5)
Supplement: Supplementary file 2 — Additional file 2. Supplementary Methods. Figure S1. Plasmid stability of serial cultures of the K. pneumoniae transconjugants RJBSI76-pV and RJBSI76-pV-hybrid. Figure S2. Homologous recombination between the IncF plasmid pRJBSI76-1 and the virulence plasmid pRJF293HA. Figure S3. Sequence alignments of the IncF plasmid pRJBSI76-1, the hybrid plasmid p1-pV-hybrid-1 and the virulence plasmid pRJF293HA. Figure S4. Genetic structure of the IncFIB plasmid pRJBSI76-1 of the clinical CRKP strain RJBSI76. Figure S5. Genetic structure of the plasmids pRJBSI76-2 and pRJBSI76-3 of the clinical CRKP strain RJBSI76. Figure S6. Sequence alignment of three predicted oriT regions. Figure S7. Plasmid stability of serial cultures of the E. coli transconjugants J53-pF-pV-hybrid and J53-pF-pV-TC2. Figure S8. Validation of the fusion event at 28-bp fusion sites. Figure S9. Schematic diagram of the mobilization of a virulence plasmid by the conjugative IncF plasmid pOX38-Gen. Figure S10. Indirect transfer of the virulence plasmid from hvKP RJF293C to CRKP RJBSI76. Figure S11. The less-frequent transfer of pRJF293C was due to increased production of extracellular polysaccharides by the donor. Figure S12. Indirect transfer of the virulence plasmid from hvKP RJF293-pF to CRKP HS11286-pKPHS2ΔoriT. Figure S13. In silico analysis of 760 conjugative plasmids of the completely sequenced K. pneumoniae in GenBank. Figure S14. Mobilization potential of 85 virulence plasmids of the completely sequenced K. pneumoniae available in GenBank. [file 13073_2021_936_MOESM2_ESM.pdf]

Supplementary data

Supplementary Methods

Figure S1. Plasmid stability of serial cultures of the *K. pneumoniae* transconjugants RJBSI76-pV and RJBSI76-pV-hybrid.

Figure S2. Homologous recombination between the IncF plasmid pRJBSI76-1 and the virulence plasmid pRJF293HA.

Figure S3. Sequence alignments of the IncF plasmid pRJBSI76-1, the hybrid plasmid p1-pV-hybrid-1 and the virulence plasmid pRJF293HA.

Figure S4. Genetic structure of the IncFIB plasmid pRJBSI76-1 of the clinical CRKP strain RJBSI76.

Figure S5. Genetic structure of the plasmids pRJBSI76-2 and pRJBSI76-3 of the clinical CRKP strain RJBSI76.

Figure S6. Sequence alignment of three predicted *oriT* regions.

Figure S7. Plasmid stability of serial cultures of the *E. coli* transconjugants J53-pF-pV-hybrid and J53-pF-pV-TC2.

Figure S8. Validation of the fusion event at 28-bp fusion sites.

Figure S9. Schematic diagram of the mobilization of the virulence plasmid by the conjugative IncF plasmid pOX38-Gen.

Figure S10. Indirect transfer of the virulence plasmid from hvKP RJF293C to CRKP RJBSI76.

Figure S11. The less-frequent transfer of pRJF293C was due to increased production of extracellular polysaccharides by the donor.

Figure S12. Indirect transfer of the virulence plasmid from hvKP RJF293-pF to CRKP HS11286-pKPHS2Δ*oriT*.

Figure S13. *In silico* analysis of 760 conjugative plasmids of the completely sequenced *K. pneumoniae* in GenBank.

Figure S14. Mobilization potential of 85 virulence plasmids of the completely sequenced *K. pneumoniae* available in GenBank.

Supplementary Methods

Construction of genetically modified strains

E. coli or *K. pneumoniae* strains containing the pKOBEG-Apr plasmid were cultured overnight in lysogeny broth (LB) medium with 50 μg/ml apramycin at 30°C and subcultured (1:100 dilution) in fresh LB broth supplemented with 0.2% L(-)-arabinose at 30°C for 2 h. Bacterial cultures were collected and prepared as electrocompetent cells. The template DNA to replace the target gene was amplified by splicing overlap extension (SOE) PCR, and the primers used are listed in Additional file 3: Table S2. Electrocompetent cells were transformed with template DNA via 0.2-cm electroporation chambers and BioRad Gene Pulser II with the following parameter settings: 200 Ω, 25 μF and 2.5 kV. The transformed cells were selected on LB plates supplemented with 50 μg/ml apramycin overnight at 37°C. The colonies were confirmed by PCR to verify the replacement of the target gene by the hygromycin resistance gene *hph* and loss of the pKOBEG-Apr plasmid. To further delete the *hph* gene, the confirmed mutant strain was prepared as electrocompetent cells and transformed with the pFLP2-Apr plasmid. The transformant was cultured overnight at 37°C on LB plates supplemented with 50 μg/ml apramycin. A single apramycin-resistant colony was then streaked onto LB plates with 6% sucrose and grown at 30°C to cure pFLP2-Apr. These colonies were confirmed by patching and PCR to detect the loss of the *hph* gene and pFLP2-Apr.

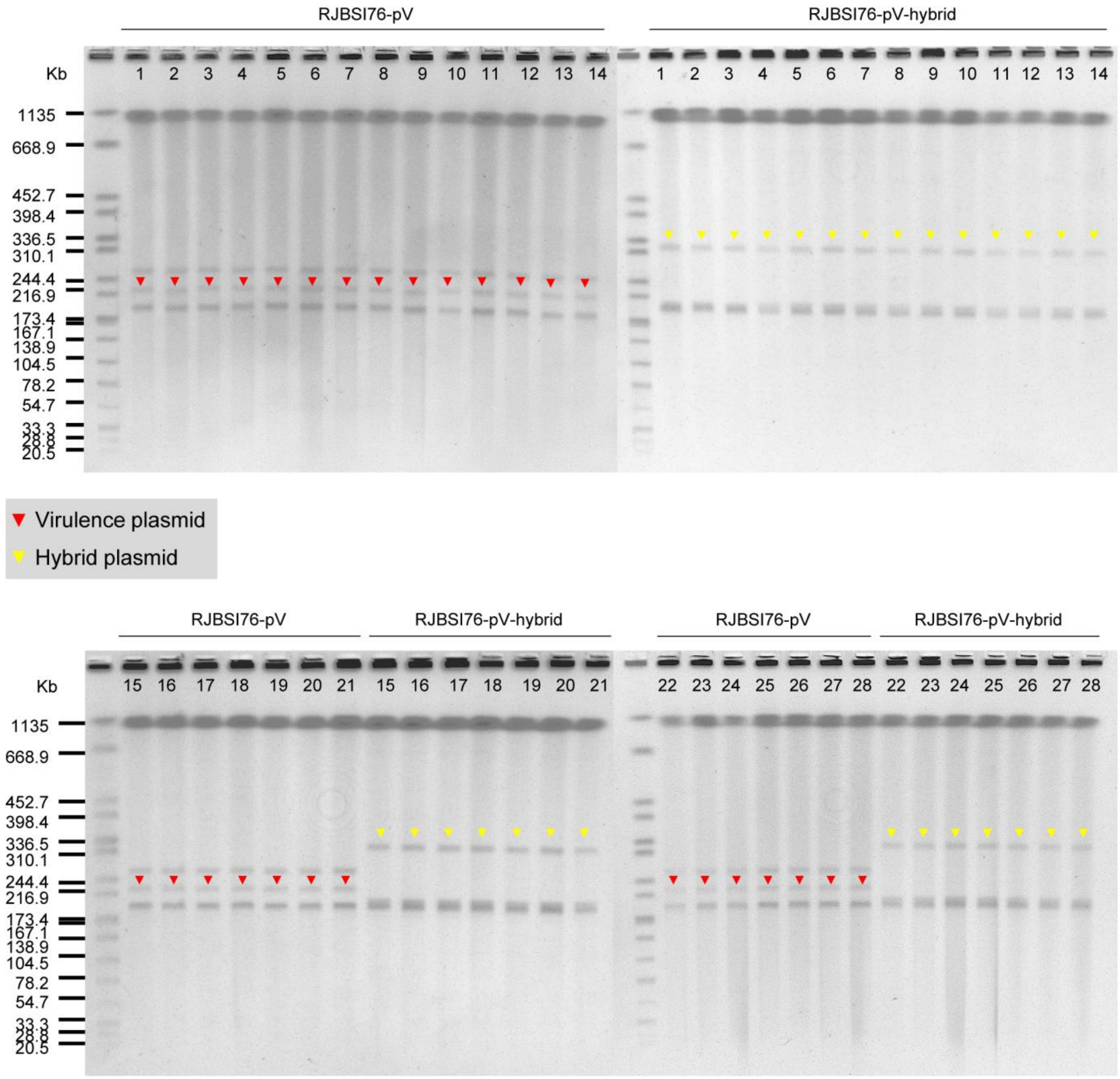

Figure S1.Plasmid stability of serial cultures of the *K. pneumoniae* transconjugants RJBSI76-pV and RJBSI76-pV-hybrid. The bacterial cultures were diluted 1:100 in 4 ml fresh LB medium every 12 h for two weeks. M represents the molecular weight marker, *Salmonella* serotype Braenderup H9812 strain. Red triangles denote the virulence plasmid pRJF293HA. Yellow triangles denote the hybrid plasmid.

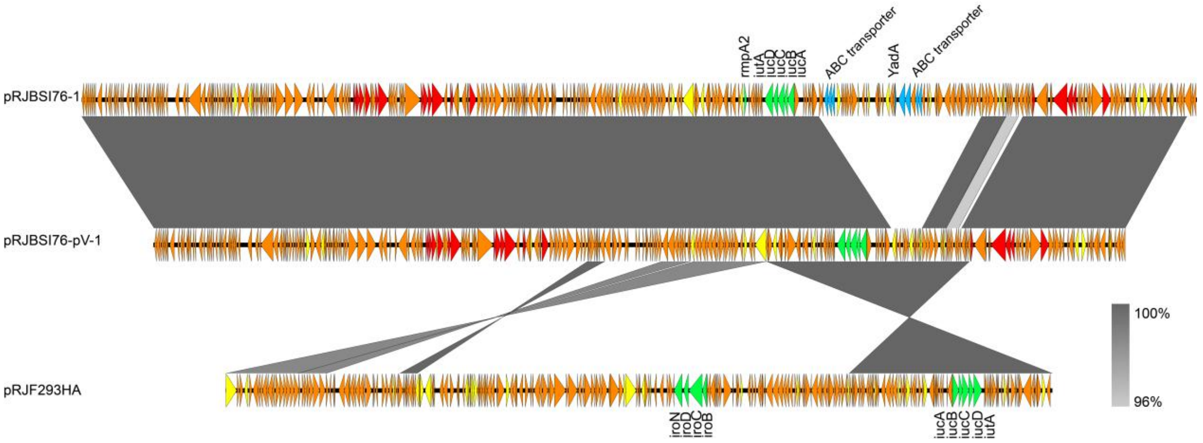

Figure S2. Homologous recombination between the IncF plasmid pRJBSI76-1 and the virulence plasmid pRJF293HA. Sequence alignments of the IncF plasmid of carbapenem-resistant *K. pneumoniae* strain RJBSI76 (pRJBSI76-1), the shorter plasmid pRJBSI76-1 in the transconjugant RJBSI76-pV (pRJBSI76-pV-1, also represented as p1') and the virulence plasmid of hypervirulent *K. pneumoniae* RJF293HA (pRJF293HA) were constructed using EasyFig [1]. Blue, ABC transporter genes. Green, virulence genes. Red, *tra* genes. Yellow, IS elements.

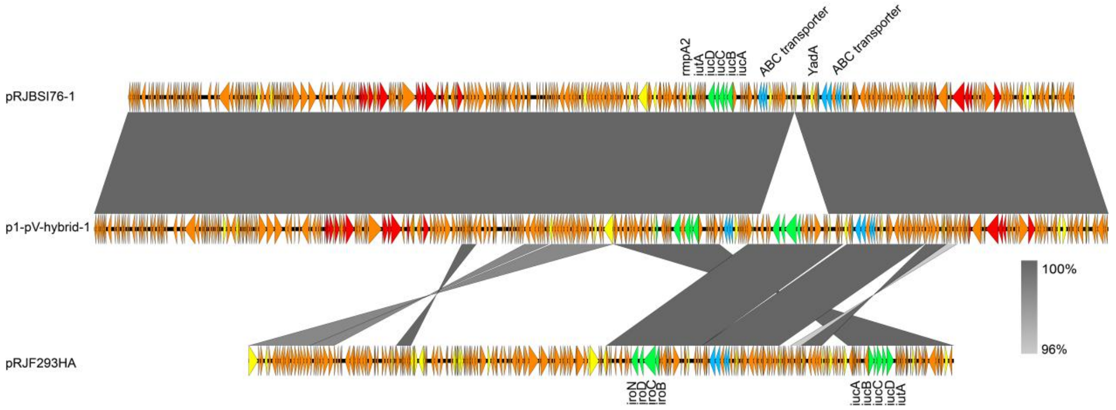

Figure S3. Sequence alignments of the IncF plasmid pRJBSI76-1, the hybrid plasmid p1-pV-hybrid-1 and the virulence plasmid pRJF293HA. pRJBSI76-1 was derived from the carbapenem-resistant *K. pneumoniae* (CRKP) RJBSI76. p1-pV-hybrid-1 was derived from *E. coli* transconjugant J53-p1-pV-hybrid-1. pRJF293HA was derived from the genetically modified hypervirulent *K. pneumoniae* (hvKP) RJF293HA. The figure was constructed using EasyFig [1]. Blue, ABC transporter genes. Green, virulence genes. Red, *tra* genes. Yellow, IS elements.

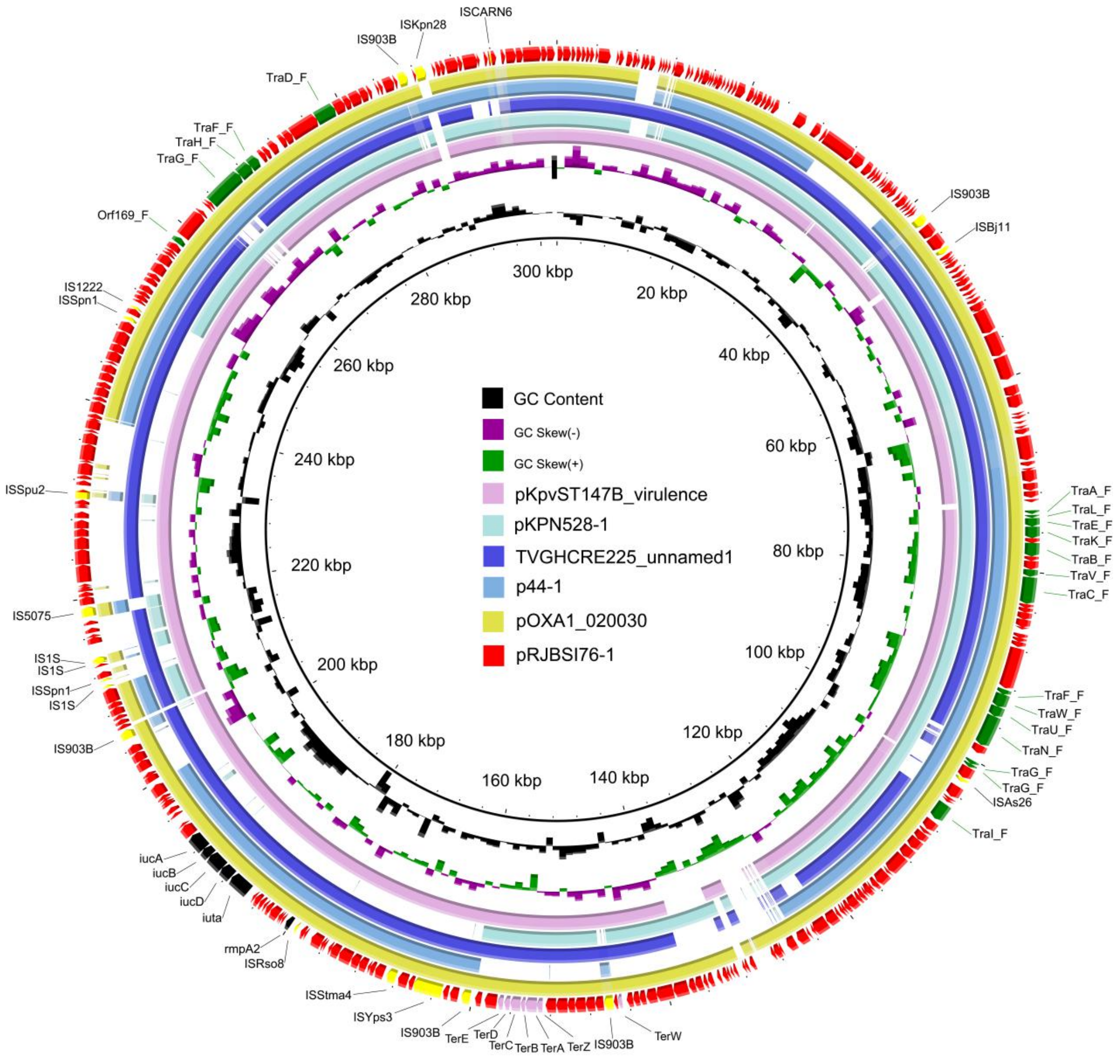

Figure S4. Genetic structure of the IncFIB plasmid pRJBSI76-1 of the clinical CRKP strain RJBSI76. pRJBSI76-1 has a length of 302,690 bp and a CG content of 46.3%. The predicted *tra* genes are shown in green and the IS elements are shown in yellow. The other five sequenced IncF plasmids sharing high sequence similarity with pRJBSI76-1 are aligned with pRJBSI76-1 using BLAST Ring Image Generator (BRIG) [2], including pKpvST147B_virulence of *K. pneumoniae* KpvST147B_SE1_1_NDM (GenBank accession no. CP040726.1), pKPN528-1 of *K. pneumoniae* KPN528 (CP020854.1), unnamed1 of *K. pneumoniae* TVGHCRE225 (CP023723.1), p44-1 of *K. pneumoniae* F44 (CP025462.1), and pOXA1_020030 of *K. pneumoniae* WCHKP020030 (CP028791.1).

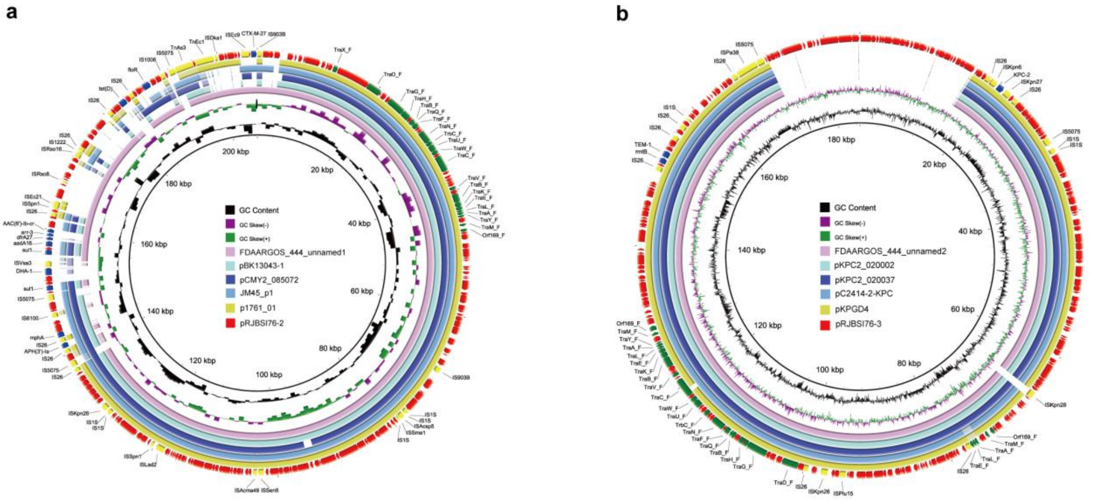

Figure S5. Genetic structure of the plasmids pRJBSI76-2 and pRJBSI76-3 of the clinical CRKP strain RJBSI76. **(a)** The IncFII$_K$/IncFIB$_K$ plasmid pRJBSI76-2 has a length of 206,263 bp and a CG content of 52.4%, and contains *tra* genes and multiple resistance genes. The aligned plasmids include unnamed1 of *K. pneumoniae* FDAARGOS_444 (GenBank accession no. CP023943.1), pBK13043-1 of *K. pneumoniae* BK13043 (CP020838.1), pCMY2_085072 of *K. pneumoniae* WCHKP7E2 (CP028804.2), p1 of *K. pneumoniae* JM45 (NC_022078.1) and p1761_01 of p1761 *K. pneumoniae* (CP039975.1). **(b)** The IncFII$_{pHN7A8}$/IncR plasmid pRJBSI76-3 contains *rmtB*, *bla*$_{KPC-2,}$ *bla*$_{TEM-1B}$ and *tra* genes, with a length of 184,748 bp and a CG content of 53.3%. The aligned plasmids include unnamed2 of *K. pneumoniae* FDAARGOS_444 (CP023942.1), pKPC2_02000 of *K. pneumoniae* WCHKP2 (CP028541.2), pKPC2_020037 of *K. pneumoniae* WCHKP020037 (CP036372.1), pC2414-2-KPC of *K. pneumoniae* C2414 (CP039820.1) and pKPGD4 of *K. pneumoniae* GD4 (CP025952.1). Alignments were performed using BRIG [2].

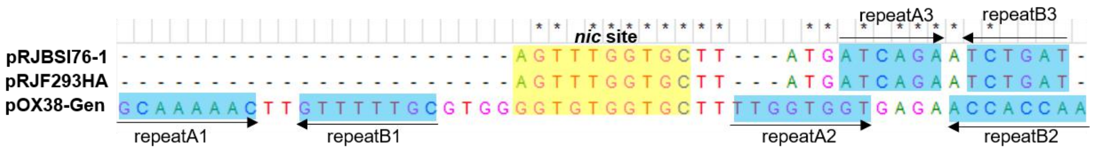

Figure S6. Sequence alignment of three predicted *oriT* regions. The blue background highlights denote the inverted repeats and the yellow background highlights denote the *nic* sites. The asterisks denote the conserved bases among three *oriT* regions on pRJBSI76-1, pRJF293HA and pOX38-Gen.

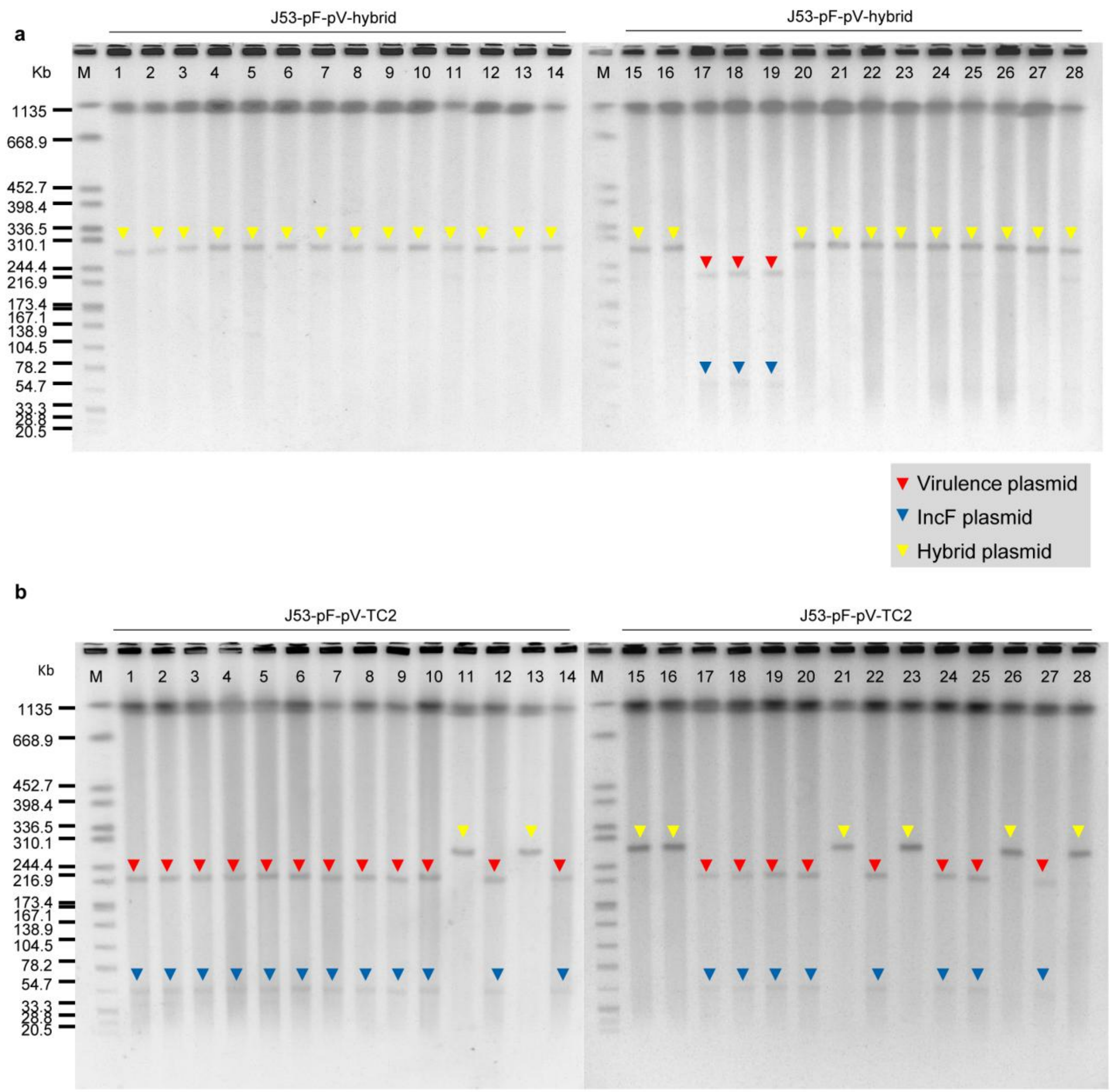

Figure S7. Plasmid stability of serial cultures of the *E. coli* transconjugants J53-pF-pV-hybrid and J53-pF-pV-TC2. M represents the molecular weight marker, *Salmonella* serotype Braenderup H9812 strain. Red triangles denote the virulence plasmid pRJF293HA. Blue triangles denote the IncF plasmid pOX38-Gen. Yellow triangles denote the hybrid plasmid.

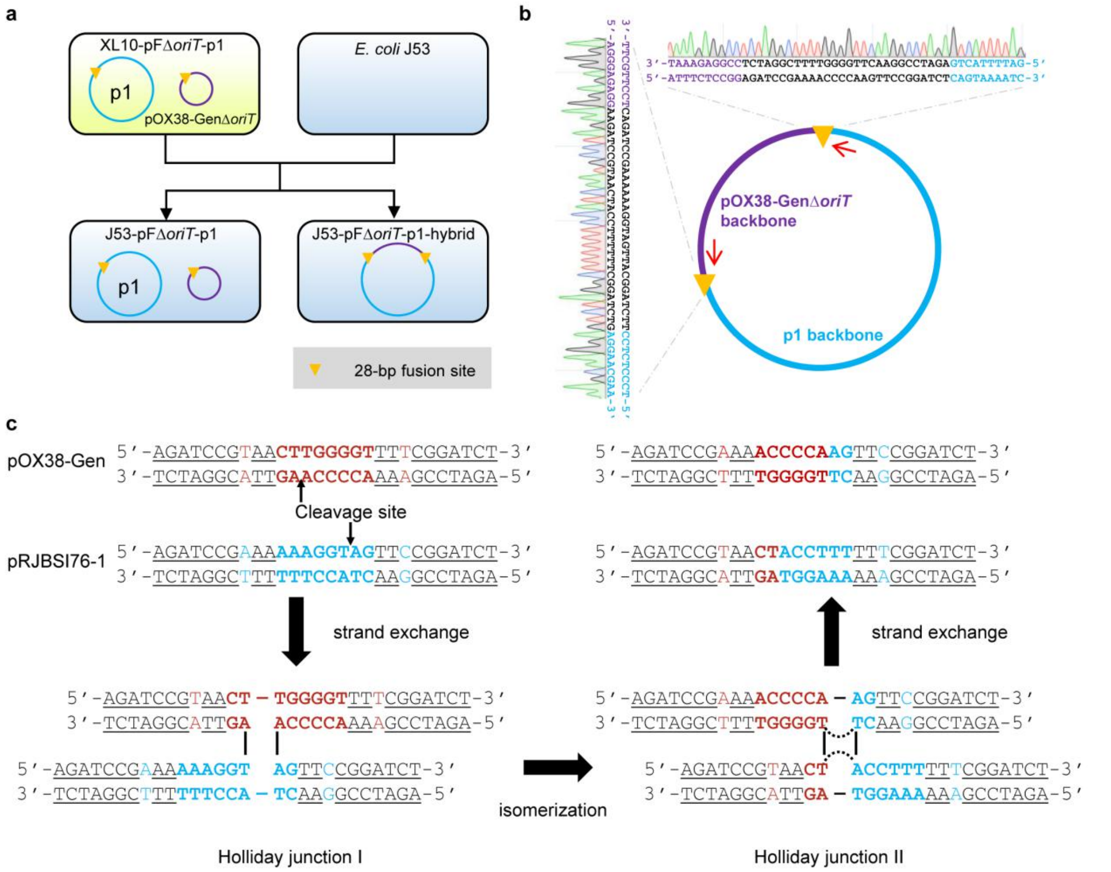

Figure S8. Validation of the fusion event at 28-bp fusion sites. **(a)** Schematic diagram of the conjugation assays between *E. coli* XL10-pFΔ*oriT*-p1 and J53. The *oriT*-deficient pOX38-Gen was mobilized under the help of p1 with or without the recombination at 28-bp fusion sites (orange triangles). **(b)** Sanger sequencing of the fusion sites between pOX38-GenΔ*oriT* and p1. The sequence of p1 was shown in blue, the sequence of pOX38-GenΔ*oriT* was shown in violet and the 28-bp sequence was shown in black. **(c)** The putative mechanism underlying the formation of the hybrid plasmid emerged from two rounds of chain cleavage and exchange between pOX38-Gen and pRJBSI76-1 within the 28-bp fusion sites, which consist of the conserved 10-bp inverted repeat sequences (underlined) and the variable 8-bp internal spacer region (bold). The arrowheads represented the supposed cleavage sites for recombination. The shared sequence was shown in black, the sequence specific to pOX38-Gen was shown in red, and the sequence specific to pRJF293HA was shown in blue.

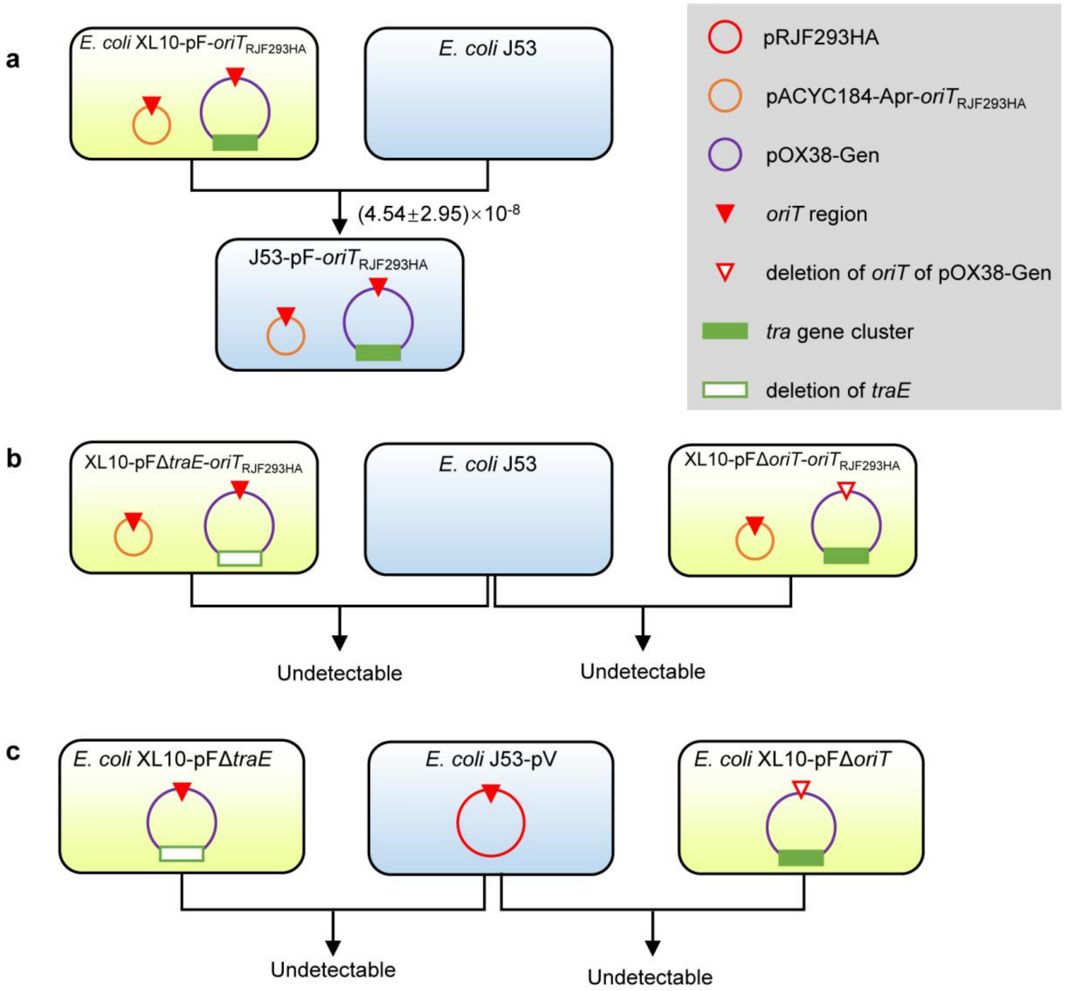

Figure S9. Schematic diagram of the mobilization of the virulence plasmid by the conjugative IncF plasmid pOX38-Gen. The green rectangle denotes the *tra* gene cluster while the hollow green rectangle denotes the deletion of *traE*. The red triangle on the plasmid pOX38-Gen denotes its *oriT* region while the hollow red triangle denotes the deletion of the *oriT* region. The red triangle on the plasmid pACYC184-Apr denotes the insertion of the *oriT* region of pRJF293HA.

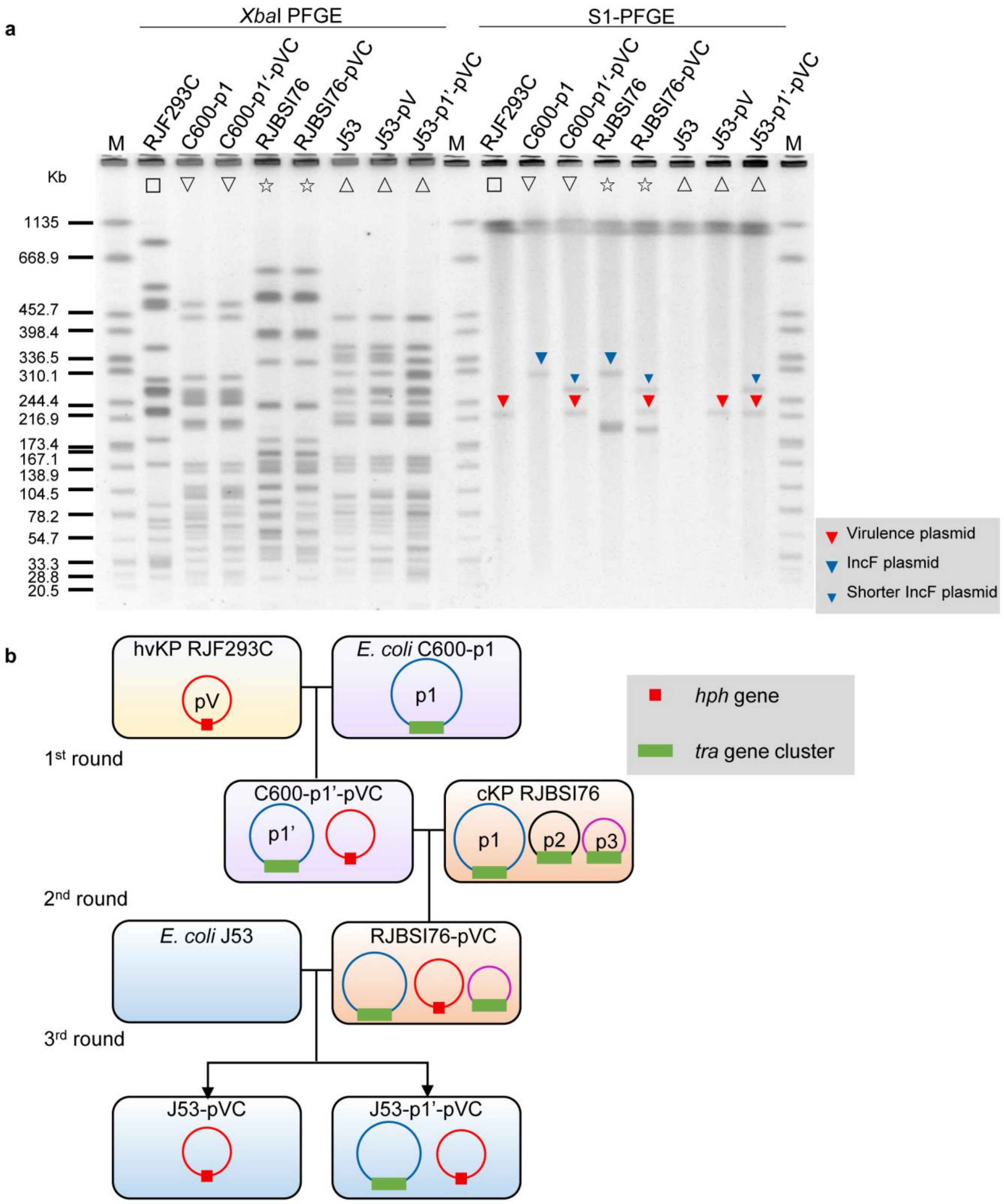

Figure S10. Indirect transfer of the virulence plasmid from hvKP RJF293C to CRKP RJBSI76. **(a)** *Xba*I PFGE and S1-PFGE of *K. pneumoniae* transconjugants and *E. coli* transconjugants as well as their parental strains. M represents the molecular weight marker, *Salmonella* serotype Braenderup H9812 strain. Red triangles denote the virulence plasmid pRJF293C. Blue triangles denote the IncF plasmid pRJBSI76-1 and its derivative. Strains with the same symbol on the PFGE image represent the progeny derived from the same parental strain. **(b)** Schematic diagram of conjugation assays. The red square denotes the *hph* tag on the virulence plasmid pRJF293C. The green rectangle denotes the *tra* gene cluster coding for T4SS on three IncF plasmids in the CRKP *strain* RJBSI76, pRJBSI76-1 (represented as p1), pRJBSI76-2 (p2) and pRJBSI76-3 (p3). The plasmid p1 in the transconjugant RJBSI76-pVC became smaller (p1').

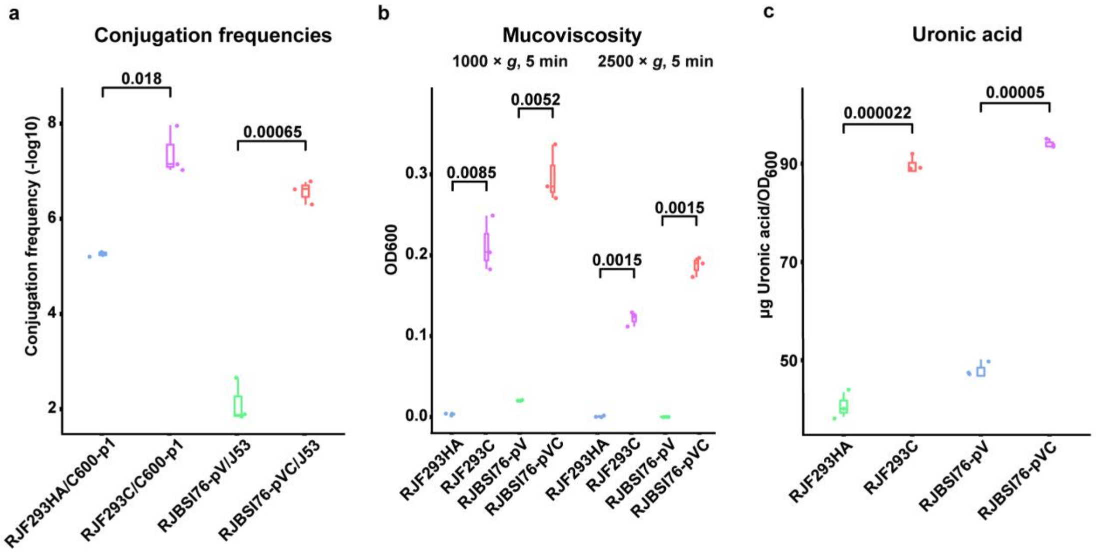

Figure S11. The less-frequent transfer of pRJF293C was due to increased production of extracellular polysaccharides by the donor. **(a)** Conjugation frequencies of mating pairs. RJF293HA and RJBSI76-pV contain the *rmpA*-deficient virulence plasmids. RJF293C and RJBSI76-pVC contain the *rmpA*-positive virulence plasmid. **(b)** Mucoviscosity and **(c)** uronic acid production level of the *K. pneumoniae* strains. The difference between the two groups in the average values was assessed by the unpaired two-sided Student's *t*-test. The central line of the Box plots represents the median, and the box outlines represent the 25% and 75% quantiles. A *P* value of less than 0.05 was considered significant. Data analysis was performed by R-4.0.3 (https://www.r-project.org/).

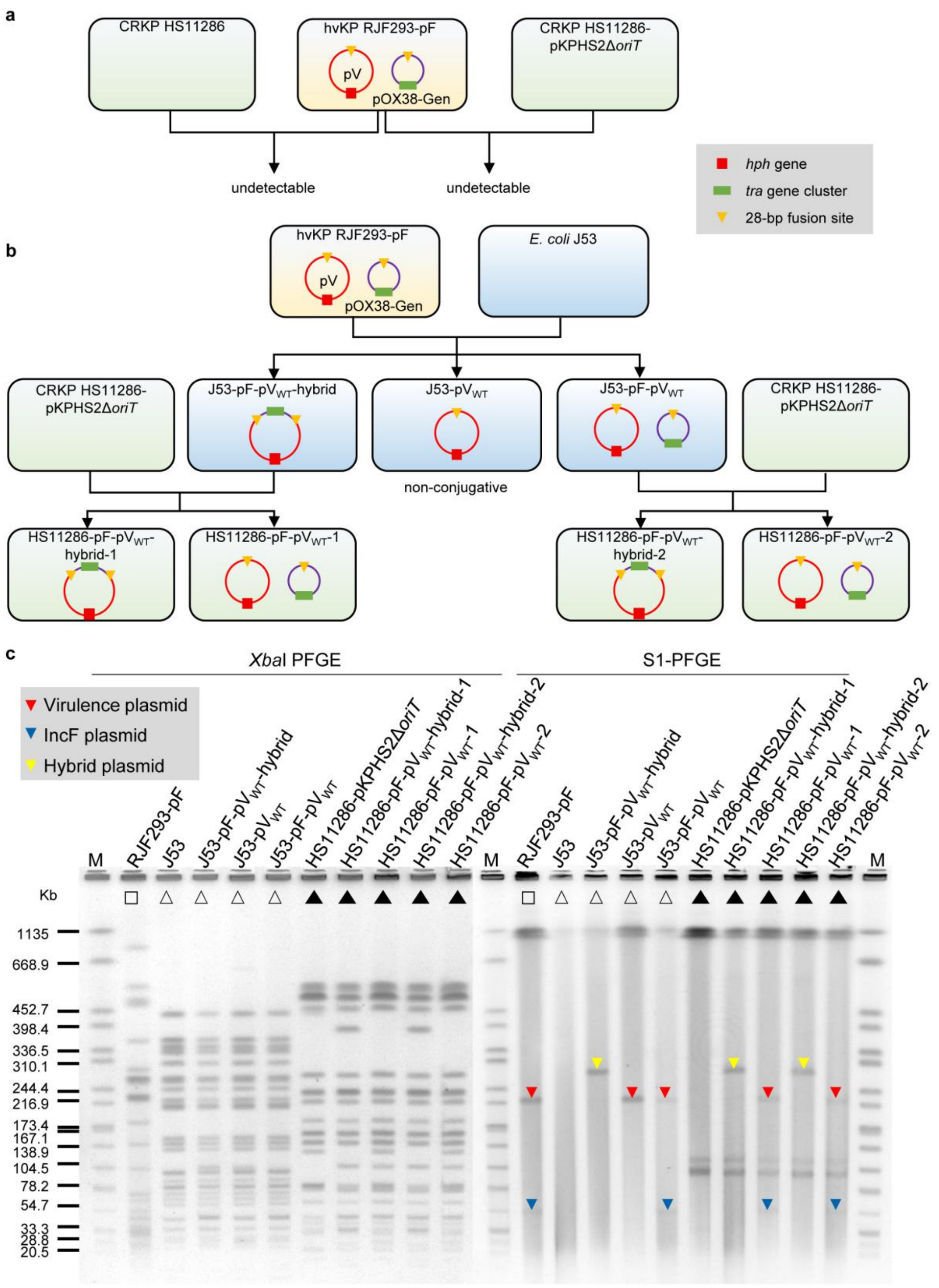

Figure S12. Indirect transfer of the virulence plasmid from hvKP RJF293-pF to CRKP HS11286-pKPHS2Δ*oriT*. **(a, b)** The schematic diagram of conjugation assays. The red square denotes the *hph* tag on the virulence plasmid pRJF293. The green rectangle denotes the *tra* gene cluster coding for T4SS on the IncF plasmid pOX38-Gen. **(c)** *Xba*I PFGE and S1-PFGE of *K. pneumoniae* transconjugants and *E. coli* transconjugants as well as their parental strains. M represents the molecular weight marker, *Salmonella* serotype Braenderup H9812 strain. Yellow triangles denote the hybrid plasmid. Red triangles denote the virulence plasmid pRJF293. Blue triangles denote the IncF plasmid pOX38-Gen. Strains with the same symbol on the PFGE image represent the progeny derived from the same parental strain.

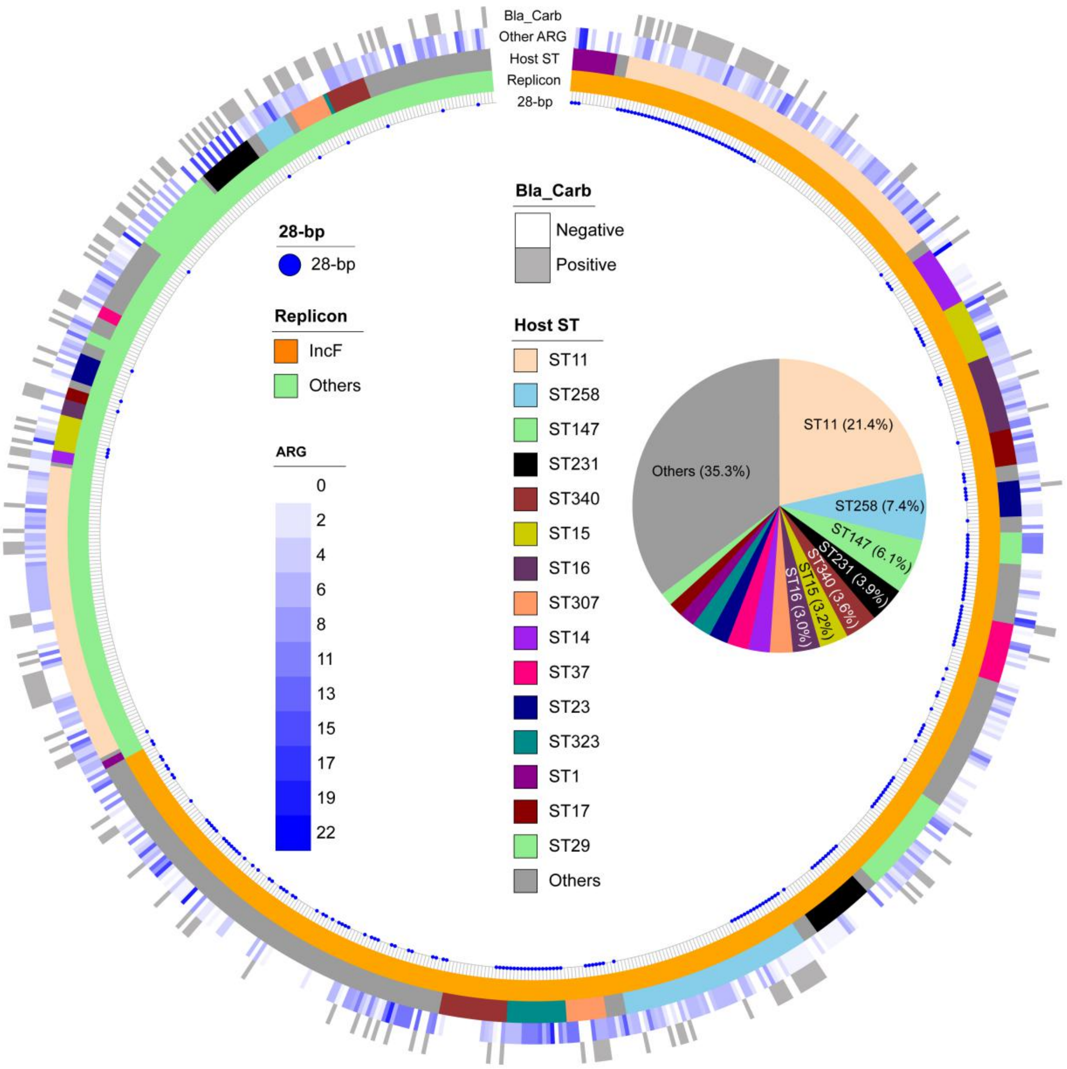

Figure S13. *In silico* analysis of 760 conjugative plasmids of the completely sequenced *K. pneumoniae* in GenBank. These conjugative plasmids containing oriTfinder-predicted *oriT* region, relaxase gene, T4CP gene and T4SS gene cluster were included. Details are listed in Table S5. Counting from the outside to the inside, the gray coloum in the first circle ('Bla_Carb') denote the presence of carbapenemase genes, including bla_{IMP-1}, bla_{IMP-4}, bla_{IMP-11}, bla_{VIM-1}, bla_{KPC-2}, bla_{KPC-3},bla_{KPC-12}, bla_{NDM-1}, bla_{NDM-4}, bla_{NDM-5}, bla_{OXA-48}, $bla_{OXA-204}$. The shade of the blue colour in the second circle ('Other ARG') denotes the number of other antimicrobial resistance genes. The varied colours in the third circle ('Host ST') and the forth circle ('Replicon') denote the types of the sequence type (ST) of the host bacteria and the plasmid replicon type, respectively. The blue dot of the fifth circle ('28-bp') denotes the distribution of the 28-bp fusion sites, which were identified by using the matchPattern function in the Biostrings R package with the specific sequence 'AGATCCGNAANNNNNNNNTTNCGGATCT'. The pie chart denotes the percentage of plasmids derived from different host STs among the 760 conjugative plasmids. The figure was constructed using iTOL [3].

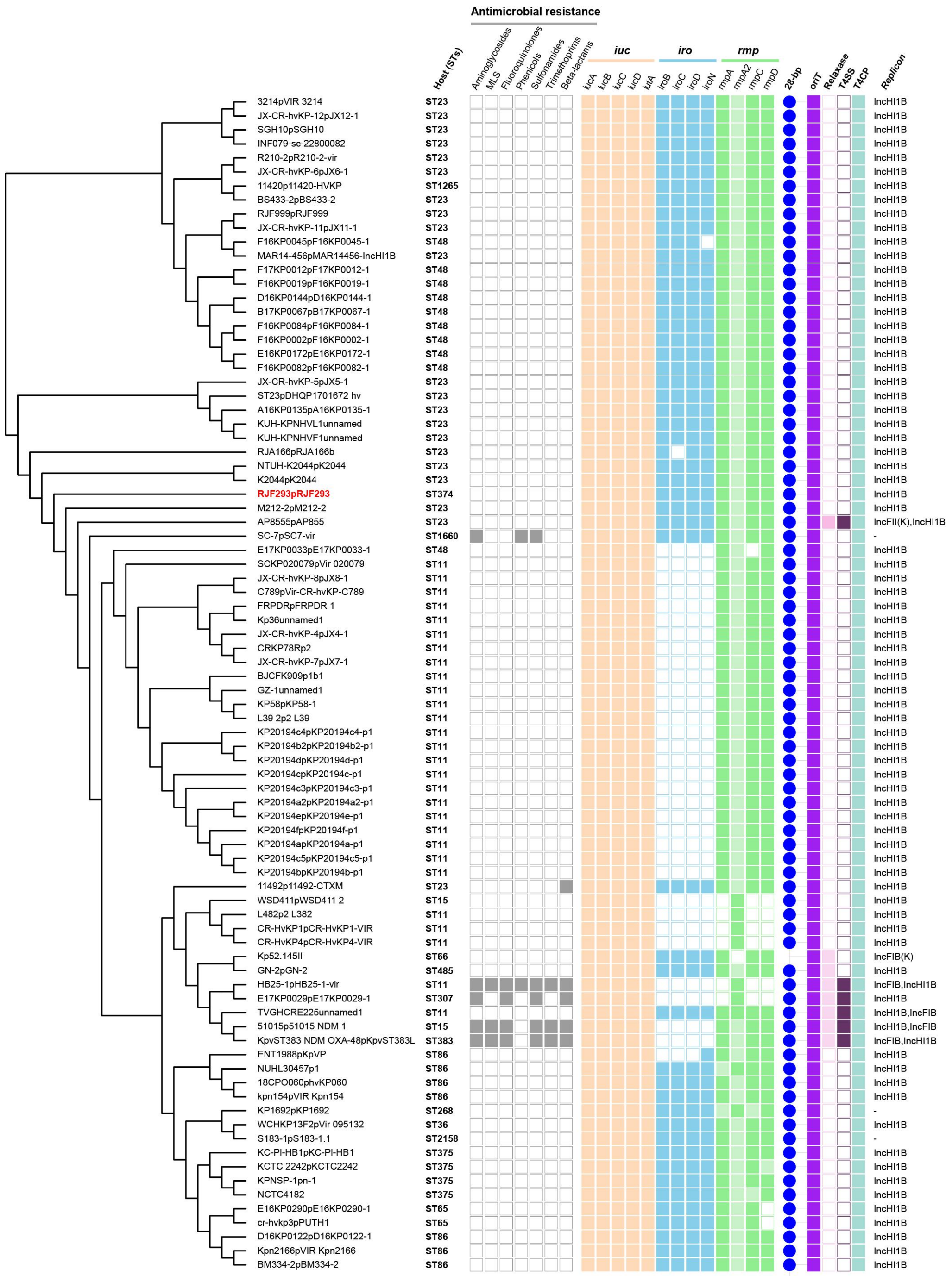

Figure S14. Mobilization potential of 85 virulence plasmids of the completely sequenced *K. pneumoniae* available in GenBank. These virulence plasmids contain the full-length *rmpA* (or *rmpA2*) and the *iuc* gene cluster. Details are listed in Table S6. The blank space denotes the absence of the corresponding gene or element. The light green space in the columns of the *rmp* cluster denotes the truncated gene. For the column of 28-bp, the absence of blue dot denotes the absence of the 28-bp fusion site. The phylogenetic tree was constructed using OrthoMCL [4]. Figure was constructed using iTOL [3].

References

1. Sullivan MJ, Petty NK, Beatson SA. Easyfig: a genome comparison visualizer. Bioinformatics. 2011;27:1009–10.

2. Alikhan N-F, Petty NK, Ben Zakour NL, Beatson SA. BLAST Ring Image Generator (BRIG): simple prokaryote genome comparisons. BMC Genomics. 2011;12:402.

3. Letunic I, Bork P. Interactive Tree Of Life (iTOL) v4: recent updates and new developments. Nucleic Acids Res. 2019;47:W256–9.

4. Li L, Stoeckert CJJ, Roos DS. OrthoMCL: identification of ortholog groups for eukaryotic genomes. Genome Res. 2003;13:2178–89.
